# Targeting PEA3 transcription factors to mitigate small cell lung cancer progression

**DOI:** 10.1038/s41388-022-02558-6

**Published:** 2022-12-13

**Authors:** David W. Shia, WooSuk Choi, Preethi Vijayaraj, Valarie Vuong, Jenna M. Sandlin, Michelle M. Lu, Adam Aziz, Caliope Marin, Cody J. Aros, Chandani Sen, Abdo Durra, Andrew J. Lund, Arunima Purkayastha, Tammy M. Rickabaugh, Thomas G. Graeber, Brigitte N. Gomperts

**Affiliations:** 1 UCLA Children’s Discovery and Innovation Institute, Mattel Children’s Hospital UCLA, Department of Pediatrics, David Geffen School of Medicine, University of California, Los Angeles, CA 90095 USA; 2 Department of Molecular Biology Interdepartmental Program, University of California, Los Angeles, CA 90095 USA; 3 UCLA Medical Scientist Training Program, David Geffen School of Medicine, University of California, Los Angeles, CA 90095 USA; 4 Department of Molecular and Medical Pharmacology, Crump Institute for Molecular Imaging, University of California, Los Angeles, CA 90095 USA; 5 Jonsson Comprehensive Cancer Center, University of California, Los Angeles, CA 90095 USA; 6 Eli and Edythe Broad Stem Cell Research Center, University of California, Los Angeles, CA 90095 USA; 7 Division of Pulmonary and Critical Care Medicine, David Geffen School of Medicine, University of California, Los Angeles, CA 90095 USA

**Keywords:** Cancer therapeutic resistance, Transcriptomics

## Abstract

Small cell lung cancer (SCLC) remains a lethal disease with a dismal overall survival rate of 6% despite promising responses to upfront combination chemotherapy. The key drivers of such rapid mortality include early metastatic dissemination in the natural course of the disease and the near guaranteed emergence of chemoresistant disease. Here, we found that we could model the regression and relapse seen in clinical SCLC in vitro. We utilized time-course resolved RNA-sequencing to globally profile transcriptome changes as SCLC cells responded to a combination of cisplatin and etoposide—the standard-of-care in SCLC. Comparisons across time points demonstrated a distinct transient transcriptional state resembling embryonic diapause. Differential gene expression analysis revealed that expression of the PEA3 transcription factors ETV4 and ETV5 were transiently upregulated in the surviving fraction of cells which we determined to be necessary for efficient clonogenic expansion following chemotherapy. The FGFR-PEA3 signaling axis guided the identification of a pan-FGFR inhibitor demonstrating in vitro and in vivo efficacy in delaying progression following combination chemotherapy, observed inhibition of phosphorylation of the FGFR adaptor FRS2 and corresponding downstream MAPK and PI3K-Akt signaling pathways. Taken together, these data nominate PEA3 transcription factors as key mediators of relapse progression in SCLC and identify a clinically actionable small molecule candidate for delaying relapse of SCLC.

## Introduction

Small cell lung cancer (SCLC) is a histological subtype of lung cancer, comprising 15–20% of lung cancer cases. It demonstrates a remarkably aggressive clinical course with early metastatic dissemination, rapid growth, and inevitable development of chemoresistant disease. Histologically, SCLC tumors are defined by their scant cytoplasm, large nuclei, and expression of neuroendocrine markers [1]. First-line standard-of-care treatment for SCLC is a combination of cisplatin and etoposide, both DNA damaging agents which are selectively toxic to rapidly dividing cells [1]. The majority of patients are ineligible for localized radiation or surgical intervention due to early widespread dissemination. Thus, the administration of systemic combination chemotherapy has remained a mainstay of treatment for SCLC. A major driver of patient mortality is the development of resistance to chemotherapy. While initial response rates are overwhelmingly positive, with rapid tumor volume reduction in a majority of patients, the development of resistant disease is near universal and often foreshadows death.

Genome-wide characterization of the mutation landscape of SCLC has only recently been accomplished, revealing near universal loss of function mutations at both *TP53* and *RB1*, key tumor suppressors with important roles across the cancer landscape [2]. The requirement for inactivation of both tumor suppressors in SCLC is further supported by genetic mouse models [3]. Notably, unlike in other forms of lung cancer, there was a lack of evidence for oncogenic driver mutations in SCLC. Kinases involved in the DNA damage response (DDR) have been uncovered as a therapeutic vulnerability and clinical development of DDR inhibitors are underway [4, 5]. Recent studies have further uncovered additional kinase targets, including a MEK5/ERK5 signaling axis and a GNAS/PKA/PP2A signaling axis [6, 7]. However, there have been no clinically approved agents to date.

While uncovering novel molecular vulnerabilities remains a key priority, uncovering mechanisms of resistance to cisplatin and etoposide is also of great importance for the field. Numerous studies have been performed to identify underlying mechanisms of resistance [8–10]. In a study of acquired resistance in patient-derived xenografts of SCLC, there was a lack of evidence of recurrent mutations associated with acquired resistance to combination chemotherapy [8].

In the current study, we sought to define the transcriptional changes occurring over the time frame of drug response and recurrence in SCLC cells with the goal of identifying regulators of this process. We identified a transient state in the population of SCLC cells following treatment with cisplatin and etoposide that was transcriptionally distinct from the initial and end state. We found this intermediate state to be enriched in transcript abundance of *ETV4* and *ETV5*. The transcription factors (TFs) encoded by these genes belong to the PEA3 subgroup of ETS-domain containing TFs and are indispensable in a number of embryonic developmental contexts [11–15]. The mouse homolog of ETV4 was previously demonstrated to play an important role in mediating distant organ metastasis in a mouse model of SCLC [16]. We discovered a key role in ETV4 and ETV5 in mediating clonogenic regrowth in SCLC following treatment with cisplatin and etoposide. We further identified an FGFR inhibitor that decreased expression of ETV4 and showed efficacy both as a single agent against SCLC and in combination with cisplatin and etoposide to slow regrowth both in vitro and in vivo.

## Results

### SCLC cellular models allow for in vitro modeling of response and relapse dynamics and acquired resistance

**Fig. 1:**
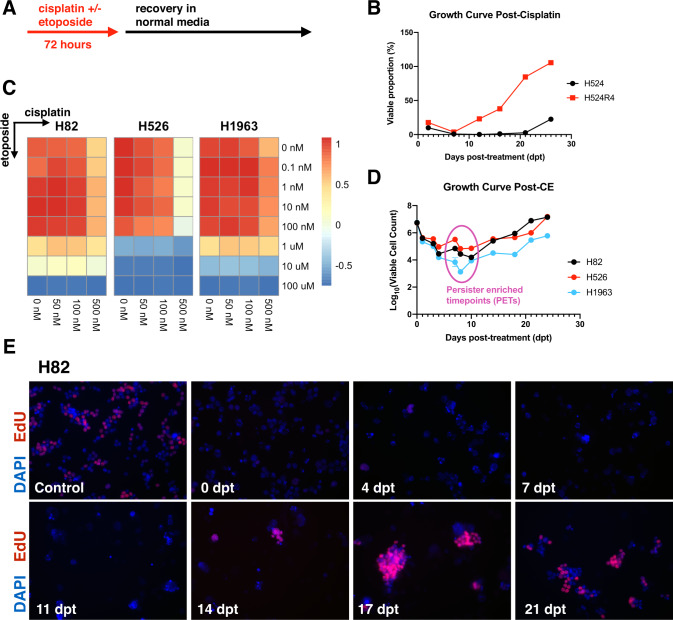
SCLC cellular models allow for in vitro modeling of acquired resistance and response and relapse dynamics. **A** Experimental timeline of chemotherapy exposure. **B** Demonstration of in vitro acquired resistance in H524 with iterative rounds of cisplatin treatment at 1 μM concentration. Viable cell number at each time point was measured in triplicate. **C** Cisplatin and etoposide dual titrations for determination of treatment concentrations. Estimated IC_50_ doses by line are as follows: cisplatin 500 nM etoposide 500 nM for H82 and H526, cisplatin 1 μM etoposide 1 μM for H1963. **D** Response and regrowth curves following 72 h treatment with combination cisplatin and etoposide at experimentally determined 72-h IC_50_ doses. Viable cell number at each time point was measured in triplicate. **E** EdU labeling at various time points following cisplatin 500 nM and etoposide 500 nM exposure for 72 h in H82.

To characterize the response to standard-of-care chemotherapy in SCLC, we pursued an in vitro cyclical treatment scheme that mimicked the administration schedule in the clinical setting. To this end, SCLC cell lines were first exposed to chemotherapy for 72-h followed by drug removal and observation (Fig. 1A). We started by treating the H524 SCLC cell line with cisplatin at 1 μM for 72-h and determined the maximal cytotoxic effect of chemotherapy occurred at seven to ten days following drug removal. At this time point, the vast majority of the culture was comprised of debris and viable clones were difficult to appreciate by light microscopy. We further monitored the culture over the next few weeks and observed the rapid expansion of surviving clones, referred to as drug-tolerant persisters (DTPs) by others across a wide spectrum of cellular cancer models and pharmacological perturbations [17–20]. We termed this resultant line H524R1. To determine whether we could model acquired resistance in vitro, we exposed H524R1 to an additional three rounds of cisplatin to generate H524R4 cells and then challenged the line with a fifth round of cisplatin. We compared the rate of regrowth in H524R4 to the parental H524 line and found that H524R4 expanded out much more rapidly following cisplatin challenge, demonstrating the ability to generate acquired resistance in vitro (Fig. 1B). Interestingly, we noted that H524R4 still demonstrated an initial response to a fifth cycle of chemotherapy, suggesting that not every clone in the population has acquired resistance through the previous cycles of chemotherapy.

**Fig. 2:**
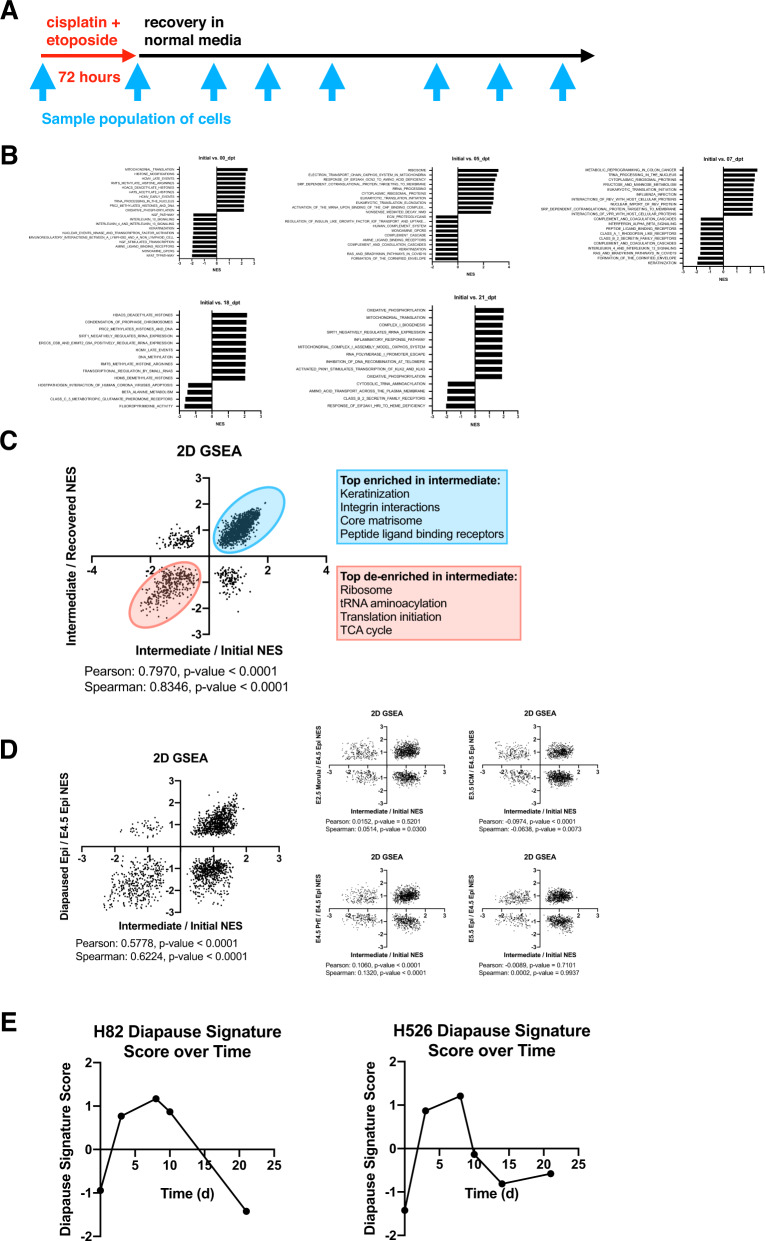
Persister-enriched time points are transcriptionally distance from initial and recovered time points and demonstrate a transient diapause-like state. **A** Timeline of sampling for RNA sequencing in cisplatin and etoposide challenge. **B** Summary of gene set enrichment analysis (GSEA) results comparing each time point to initial. Positive normalized enrichment scores (NES) represent gene sets enriched in the initial time point. **C** Two-dimensional GSEA (2D GSEA) comparing the intermediate to initial and intermediate to recovered comparisons and demonstrating a high positive correlation. **D** 2D GSEA comparing the intermediate to initial with various stages of murine embryo development. **E** Diapause gene signature scoring of each time point.

We then expanded this approach to a combination of cisplatin and etoposide to most accurately model the clinical treatment for SCLC. We performed dual dose titrations with cisplatin and etoposide across three SCLC cell lines and found no evidence of synergistic cytotoxicity across the ranges of doses tested (Fig. 1C, Supplementary Fig. 1A), in line with previous studies [4]. Using three SCLC cell lines (H82, H526, H1963), we determined the 72-h IC_50_ doses to be optimal in reducing absolute viable cell numbers by three to four orders of magnitude from the starting population while also exhibiting rapid expansion of persisting clones by 18 to 21 days following drug removal (Fig. 1D, Supplementary Fig. 1B, C). Similar to our initial studies with single-agent cisplatin, we determined the maximal cytotoxic effect to be seven to ten days after drug removal, which we termed the persister-enriched time point (PET) [2]. We further performed EdU labeling at various time points after treatment with cisplatin and etoposide and found that the proportion of EdU-positive cells was abruptly reduced to near-undetectable levels for the first 14 days following drug removal (Fig. 1E, Supplementary Fig. 1D), consistent with our flow cytometry quantifications. We next sought to define the underlying molecular features distinguishing these DTPs from the starting cell population. To this end, we longitudinally sampled cells at various time points along the time course for RNA-sequencing (Fig. 2A). RNA-sequencing was performed on two of the three time courses (H82 and H526 cell lines).

### Persister-enriched time points are transcriptionally distinct from initial and recovered time points and demonstrate a transient diapause-like state

To identify gene expression changes induced by combination chemotherapy, we performed differential gene expression analysis of each time point compared to the initial time point prior to chemotherapy exposure. We used distance clustering to exclude outlier time points from this analysis (Supplementary Fig. 2A). Using the list of differentially expressed genes (DEGs) from each comparison, we implemented gene set enrichment analysis (GSEA) to identify biological processes and pathways that were enriched at each time point (Fig. 2B). We noted consistent de-enrichment of rRNA processing, ribosome, and translation initiation terms at 0-, 5-, and 7-days post-treatment, in line with the known effects of cisplatin administration [21, 22]. We noted a consistent enrichment of keratinization, core matrisome, complement and coagulation cascades, and amine ligand binding receptors terms at the same time points. A number of recent studies across various cancer models have documented the transient nature of DTP-specific gene expression [18, 19]. We hypothesized that the DTP gene expression signature at our defined PET of 7-days post-treatment was also transient in nature. Principal component analysis of each dataset by cell line demonstrated a separation of the intermediate time points (Supplementary Fig. 2B). To further assess this, we first defined the 7-days post-treatment as intermediate and the 21-days post-treatment as recovered. We then plotted the normalized enrichment scores for each gene ontology term derived from GSEA analyses from two comparisons: (1) initial to intermediate, and (2) intermediate to recovered [23]. We found a Pearson correlation of 0.797 and a Spearman correlation of 0.8346 between the initial and recovered time points, demonstrating a high degree of similarity pre- and post-treatment (Fig. 2C).

Embryonic diapause is a state of developmental suspension which embryos can undergo in response to environmental challenge [24, 25]. As several recent studies have begun to uncover similarities of cancer cell drug resistant states to embryonic diapause, we sought to evaluate whether SCLC DTPs also adopted a diapause-like state. We compared our initial to intermediate DEGs to that of the various stages of mouse embryo development and found significant correlation when we compared initial to intermediate against a comparison of E4.5 epiblast against diapaused embryos (Fig. 2D) [26]. We utilized the DEGs (Supplementary Table 1) from the E4.5 epiblast compared to diapaused embryos to generate a gene signature score for each time point and found the diapause signature score was increased at PETs in a transient manner (Fig. 2E), thus supporting the transient nature of this diapause-like transcriptional state.

### Drug-tolerant persisters are enriched in expression of PEA3 transcription factors ETV4 and ETV5

**Fig. 3:**
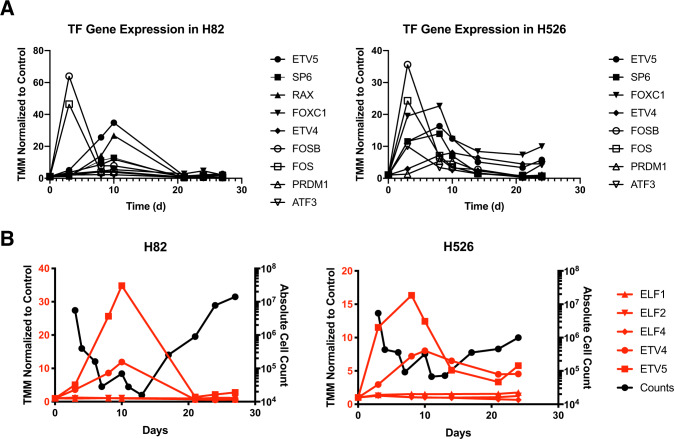
Drug-tolerant persisters are enriched in expression of PEA3 transcription factors ETV4 and ETV5. **A** Expression of top intermediate-enriched transcription factors in each cell line dataset. **B** Expression of PEA3 and ELF subgroups of transcription factors plotted against viable cell counts at each time point.

We next narrowed our gene expression analyses to focus in on the transcription factor (TF) families of genes. We reasoned that the underlying transcriptional signature in SCLC DTPs was either driven by drug-induced changes in TF activity or was inherent to a rare subpopulation of cells. We found that consistently between datasets from both cell lines, there was a significant increase in the expression of two TFs, ETV4, and ETV5 (Fig. 3A). When expression of ETV4 and ETV5 was plotted against the growth curves for each cell line, we found that the expression of both TFs peaked at the time points enriched in DTPs and exhibited a relative decrease as persistent clones expanded out in the latter portion of each time course (Fig. 3B). Both ETV4 and ETV5, alongside ETV1, belong to the PEA3 subfamily of transcription factors, which itself is a member of the ETS family of transcription factors. There are a total of 29 human genes encoding ETS family transcription factors, all of which share a highly conserved ETS DNA binding domain [27]. ETV4 and ETV5 have been previously implicated in mediating progression of disease in a variety of cancer systems [27, 28]. These TFs have been most classically studied in the context of prostate cancer and melanoma, where there have been documented examples of translocation and amplification events resulting in overexpression of PEA3 subfamily members [29]. Previous literature has suggested redundant function between *ETV4* and *ETV5*, but further study is warranted to determine whether such redundancy is constitutive or context dependent. To gain a better understanding of the possible relevance of *ETV4* and *ETV5* in the context of SCLC biology, we first examined global gene expression databases. We performed Pearson correlation analysis on the gene expression of all members of the ETS family of transcription factors in both SCLC cell line and primary tumor datasets [2]. We found that among all ETS family transcription factors, *ETV4* and *ETV5* consistently demonstrated the highest Pearson correlation coefficient between each other in both the cell line and primary tumor datasets (Supplementary Fig. 3A, B). We queried the DepMap database to determine whether perturbation of *ETV4* and *ETV5* at both the gene and transcript level could be detrimental to SCLC cell viability. Knockdown or knockout of *ETV4* or *ETV5* individually did not result in decreased viability in the context of global screens (Supplementary Fig. 3C, D). Given the enrichment of both *ETV4* and *ETV5* expression in DTPs and the lack of evidence of any single gene perturbation lethality, we sought to directly perturb both *ETV4* and *ETV5* to test their role in mediating survival and expansion of SCLC DTPs following combination chemotherapy.

### ETV4 and ETV5 are regulators of clonogenic regrowth following combination chemotherapy in SCLC

**Fig. 4:**
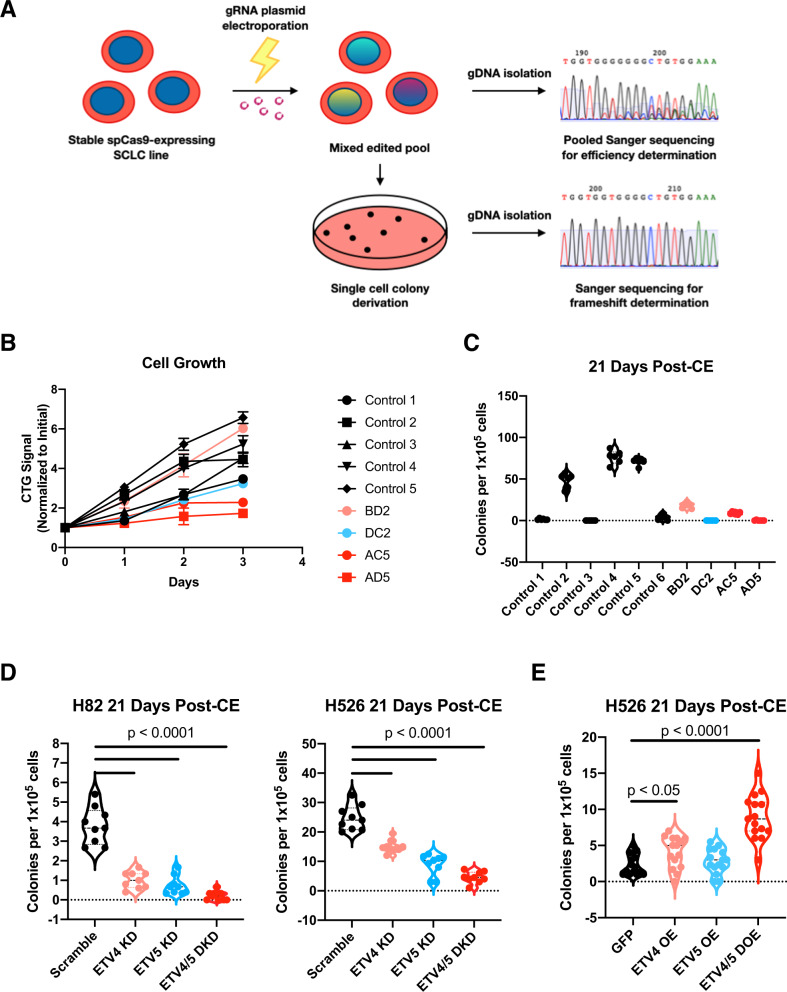
ETV4 and ETV5 are regulators of regrowth following combination chemotherapy in SCLC. **A** Schematic demonstrating workflow for generation of loss-of-function mutant lines. **B** Cellular growth curves of monoclonal sublines derived from H526 parental line comparing wild-type lines to mutant lines generated from CRISPR-Cas9 by CellTiter-Glo. Each time point was measured in quadruplicate. **C** Clonogenic regrowth assay in monoclonal sublines following challenge with combination 500 nM cisplatin and etoposide for 72 h. **D** Clonogenic regrowth assay in stably-transduced H82 and H526 lines expressing the following shRNA: scrambled, ETV4, ETV5, ETV4 and ETV5. Cells were treated with 500 nM cisplatin and etoposide for 72 h and then seeded in 1% methylcellulose for quantification of clonogenic regrowth. Non-parametric *t*-test was used to determine statistical significance between groups. **E** Clonogenic regrowth assay in stably-transduced H526 lines expressing the following cDNA: GFP, ETV4, ETV5, ETV4, and ETV5. As above, cells were treated with 500 nM cisplatin and etoposide for 72 h and then seeded in 1% methylcellulose for quantification of clonogenic regrowth. Non-parametric t-test was used to determine statistical significance between groups.

To evaluate the necessity of *ETV4* and *ETV5* in progression of SCLC following cisplatin + etoposide, we used CRISPR-Cas9 to generate double knockout mutant lines (Fig. 4A). We targeted exons common to multiple known transcript splice isoforms and derived monoclonal lines with stable frameshift mutations confirmed by Sanger sequencing that resulted in a complete loss of function (Supplementary Fig. 4A, B). Through this method, we were able to generate two lines with homozygous frameshift mutations (FM) at the desired exons in both *ETV4* and *ETV5*, referred to as lines AC5 and AD5. We identified two additional lines, one with homozygous FM at *ETV4* and heterozygous FM at *ETV5* referred to as BD2 and another with heterozygous FM at *ETV4* and homozygous FM at *ETV5* referred to as DC2. To assess the specificity of the spacer sequences used in our gene edited clones we assessed two predicted potential protein-coding off-target sites located in the exon regions of the genes *ENO2* and *WDR93*. We found no evidence of off-target editing at either of these sites across all generated mutant lines (Supplementary Fig. 4C). We generated unedited monoclonal control lines in parallel using a gRNA plasmid with an empty spacer region and verified the lack of any edit at *ETV4* and *ETV5* in each line. We first assayed the growth rate of control and mutant lines and found that despite an appreciable degree of variation in growth rates between individual control lines, AC5 and AD5 trended towards a reduced growth rate compared to controls but did not reach statistical thresholds (Mann–Whitney, *p* = 0.0952) (Fig. 4B). We then proceeded to assess the regrowth potential of each line following challenge with combination chemotherapy. Strikingly, we found a significant degree of variation amongst the six unedited, control lines (Fig. 4C). While three control lines tested demonstrated robust clonogenic regrowth ranging from 50 to 90 colonies per 100,000 cells treated, the remaining three had little to no colony formation. We found that our two *ETV4*^*FM/FM*^*; ETV5*^*FM/FM*^ lines averaged 10 and <1 colonies per 100,000 cells treated, respectively (Fig. 4C). We reasoned that underlying transient transcriptional fluctuation could be an underlying driver of the drug response variation in our control lines, as has been noted by others [17]. While the results of our CRISPR-Cas9 studies were supportive of a role for *ETV4* and *ETV5* in SCLC persistence, our observed single cell variability precluded a statistically significant conclusion. To circumvent the issue of underlying single cell heterogeneity, we turned to a population-based approach and utilized shRNA to mediate *ETV4* and *ETV5* knockdown at the transcript level. We generated stably expressing shRNA lines from H82 and H526 parental lines and confirmed knockdown efficiency by qPCR (Supplementary Fig. 4D). We found no statistically significant difference in growth rate between the resultant lines over a 72 h time course (Supplementary Fig. 4F). We then performed clonogenic assays following challenge with combination chemotherapy and found a statistically significant decrease in the number of colonies in the double knock down group compared to the control consistently in both H82 and H526 cell lines. We found that while single knockdowns of ETV4 or ETV5 produced statistically significant decreases in persisting colony formation, the combination of the two produced a higher magnitude of decrease consistently between both cell lines (Fig. 4D). We further generated lines to overexpress ETV4 and ETV5 individually and in combination (Supplementary Fig. 4E) and found that the overexpression of both ETV4 and ETV5 improved clonogenic regrowth capacity without significant effects on the basal growth rates of the line (Supplementary Fig. 4G, Fig. 4E). Thus, we conclude that expression of both ETV4 and ETV5 are required for full SCLC persistence in response to combination chemotherapy. Furthermore, overexpression of both appears to be sufficient to increased clonogenic regrowth capacity and targeting their regulation could be useful for the treatment of SCLC.

### In vitro evaluation of pan-FGFR inhibitors in preventing recurrence following combination chemotherapy

**Fig. 5:**
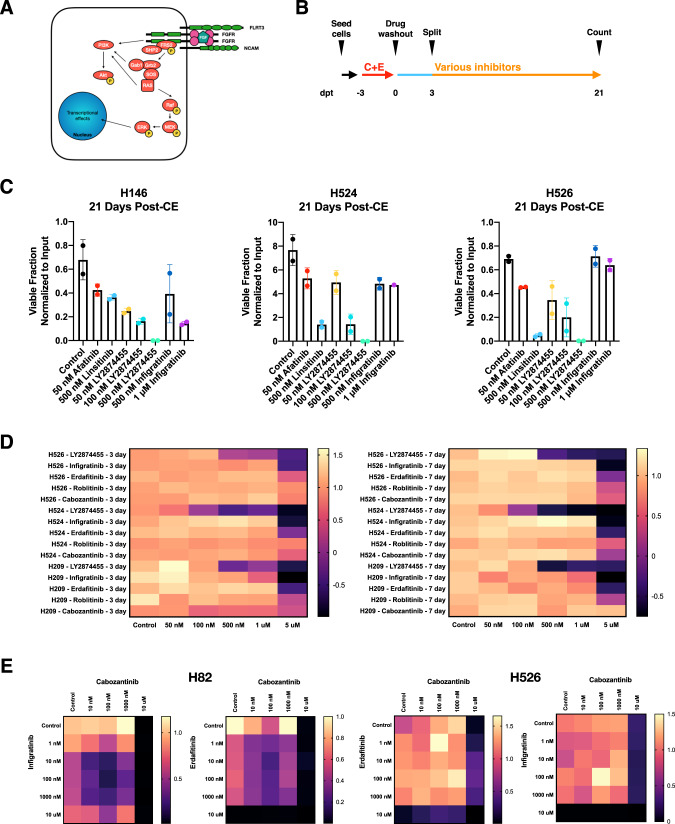
In vitro evaluation of pan-FGFR inhibitors in preventing recurrence following combination chemotherapy. **A** Generalized FGFR intracellular signaling pathway. **B** Experimental scheme for kinase inhibitor evaluation. **C** Viable cell counts at 21 days following 500 nM cisplatin and etoposide challenge across three SCLC cell lines: H146, H524, and H526. **D** Evaluation of single agent cytotoxicity of pan-FGFR inhibitors at 3 day and 7 day time points across three SCLC cell lines: H209, H524, and H526. **E** Dual titrations of pan-FGFR inhibitors infigratininb and erdafitinib against cabozantinib in H82 (left) and H526 (right).

Given that both ETV4 and ETV5 are known to be downstream transcriptional effectors of fibroblast growth factor receptor (FGFR signaling), we next turned our focus to identifying therapeutically actionable targets in this pathway (Fig. 5A) that could be implemented in a clinical setting. While many FGFR ligands exist, the FGFR family of receptors is limited to four members. We identified several commercially available pan-FGFR inhibitors that have either been approved or are under clinical development (Supplementary Fig. 5A). We performed a chemotherapy challenge assay with cisplatin and etoposide as previously described (Fig. 1A) across three different SCLC cell lines (H146, H524, and H526) and split the resultant cultures into different conditions to test the efficacy of pan-FGFR inhibition in preventing cell regrowth (Fig. 5B). We found that the pan-FGFR inhibitor LY2874455 demonstrated a dose-dependent decrease in the number of viable clones able to expand out at 21 days post-treatment consistently across all three cell lines tested, with no detectable viable cells by flow cytometry at the highest tested concentration of 500 nM (Fig. 5C). Infigratinib, an inhibitor of FGFR1/2/3, demonstrated inconsistent results across the different cell lines tested. We next sought to determine whether the anti-proliferative effect of LY2874455 was specific to SCLC DTP clones. We titrated LY2874455, infigratinib, an additional pan-FGFR inhibitor erdafitinib, and a specific FGFR4 inhibitor roblitinib across a dose range up to 5 μM for either three or seven days and found that LY2874455 demonstrated significant in vitro cytotoxicity against all lines tested (Fig. 5D) suggesting that LY2874455 cytotoxicity was not unique to the DTP clones. Unlike the other FGFR inhibitors tested, LY2874455 is known to also have inhibitor activity against VEGFR2 [30]. We, therefore, performed dual titration assays between pan-FGFR inhibitors infigratinib and erdafitinib against either a VEGFR2-specific inhibitor cabozantinib or a pan-VEGFR inhibitor lucitanib, but found conflicting evidence. In H82, there was consistent evidence of synergistic decreases in cell viability when pan-FGFR inhibitor was combined with both VEGFR inhibitors tested. However, in H526 there was no evidence of synergy with these combinations of inhibitors (Fig. 5E, Supplementary Fig. 5B). Thus, it is possible that in H526, LY2874455 may also interact with other pathways outside of FGFR and VEGFR that account for its efficacy. In sum, these results identify LY2874455, a previously described pan-FGFR inhibitor, as a small molecule with the ability to reduce regrowth across a panel of SCLC cell lines with both upfront treatment and following combination chemotherapy. Further work will need to be done to determine the full inhibitory spectrum of LY2874455.

### LY2874455 is a unique FGFR inhibitor that demonstrates inhibitory activity of downstream FRS2, MAPK, PI3K-Akt signaling pathways

**Fig. 6:**
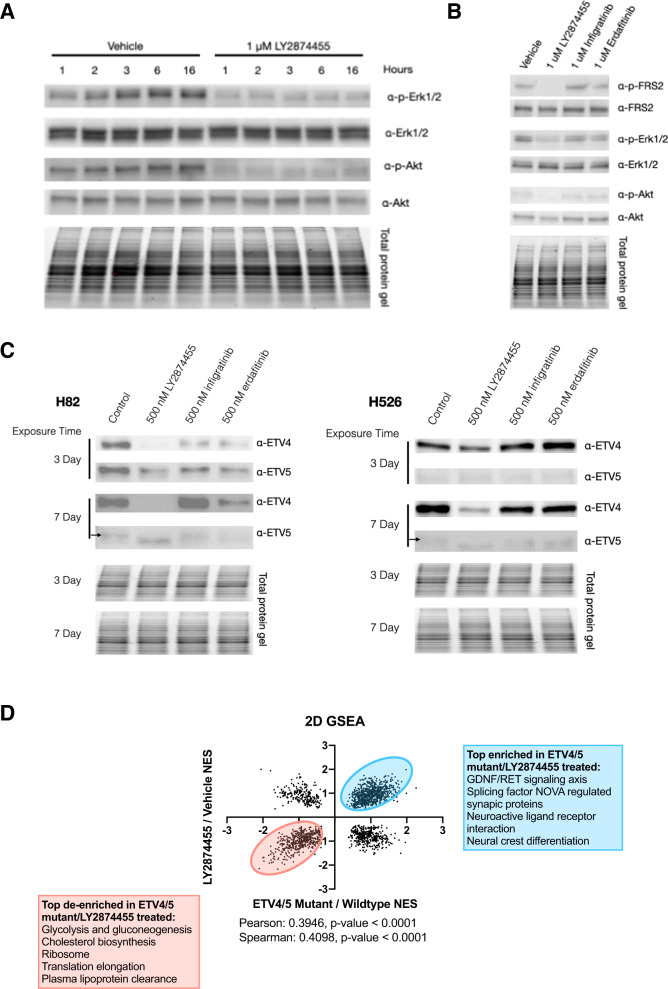
LY2874455 alone demonstrates inhibitory activity of downstream MAPK and PI3K-Akt signaling pathways. **A** Time course analysis of phosphorylation of Erk1/2 (T202/Y204) and Akt (S473) following exposure to 1 μM LY2874455 in H526. **B** Immunoblot evaluation of phosphorylation in FRS2 (Y196), Erk1/2 (T202/Y204), and Akt (S473) following 16 h treatment with 1 μM of each pan-FGFR inhibitor: LY2874455, infigratinib, and erdafitinib in H526. **C** Immunoblot evaluation of ETV4 and ETV5 protein expression following 3 and 7 days of exposure to the pan-FGFR inhibitors LY2874455, infigratinib, and erdafitinib in H82 and H526. **D** 2D GSEA comparing H526 ETV4/5 double mutant to H526 wildtype and H526 LY2874455 exposed to vehicle exposed comparisons to one another.

To begin to mechanistically understand the effect of LY2874455 on SCLC cells, we turned to immunoblot assays. We hypothesized that LY2874455 acted in part through inhibition of the FGFR signaling cascade (Fig. 5A). We first characterized the kinetics of LY2874455 inhibition in SCLC cells. We treated SCLC cells with either vehicle or 1 μM LY2874455 and collected samples at various time points up to 16 h. We found that treatment with LY2874455 blocked downstream Erk1/2 phosphorylation at T202/204 and Akt phosphorylation at S473 within 1 h of drug administration and this effect was maintained up to 16 h of treatment (Fig. 6A). At 500 nM of LY2874455, we found reduced phosphorylation inhibition (Supplementary Fig. 6A). We then wondered whether other pan-FGFR inhibitors could achieve similar signaling perturbations in SCLC cells. To this end, we treated SCLC cells with either vehicle, LY2874455, infigratinib, or erdafitinib for 16 h at 1 μM each and collected protein lysates for immunoblot analysis. We first assessed phosphorylation of FRS2, a known signal transducing adaptor protein in intracellular FGFR signaling. We found that the lysates of LY2874455-treated cells had markedly reduced levels of phospho-FRS2 at Y196 (Fig. 6B). We further assessed the phosphorylation states of downstream Erk1/2 and Akt and found that only LY2874455 reduced levels of phospho-Erk1/2 at T202/Y204 and phospho-Akt at S473 (Fig. 6B). Interestingly, we found that both infigratinib and erdafitinib failed to block signaling both at the upstream level of FRS2 and at the more downstream levels of both the MAPK and PI3K-Akt signaling pathways (Fig. 6A). While we tried to blot directly for phosphorylated forms of FGFR, we were unable to appreciate any convincing blotting. This raises the possibility that the cytotoxic activity of LY2874455 in SCLC cells could also be due to inhibition of other yet to be identified kinases. FRS2 has been found to mediate intracellular signaling through other receptor tyrosine kinases, including ALK and TrkA [31, 32]. To determine whether treatment with LY2874455 could affect downstream expression of ETV4 and ETV5 protein, we immunoblotted for these transcription factors. We found no change in ETV4 protein expression with short-term LY2874455 treatment on the order of hours, but when cells were treated with LY2874455 over and extended time course (3 and 7 days), we were able to observe a decrease in ETV4 and ETV5 protein expression (Fig. 6C). We observed no such effect from long-term infigratinib or erdafitinib treatment. Given our observation that overexpression of both *ETV4* and *ETV5* was sufficient to increase clonogenic regrowth following chemotherapy, we sought to evaluate whether such overexpression could rescue clonogenic regrowth in the setting of LY2874455 treatment. To do so, we exposed the four overexpression lines used previously to 500 nM cisplatin and etoposide for 72 h as before. We then seeded these lines in semisolid methylcellulose containing a final concentration of 500 nM LY2874455. We found no significant difference in the clonogenic regrowth between the GFP-overexpressing control line and the ETV4 and ETV5-overexpression lines in this setting (Supplementary Fig. 6B), suggesting that ETV4 and ETV5 are not the only downstream targets of LY2874455 inhibition. To determine if LY2874455 treatment elicited similar effects as loss-of-function *ETV4* and *ETV5* mutations, we performed 2D GSEA comparisons between our previously generated mutant and control lines and H526 cultures exposed to either vehicle or 500 nM LY2874455. We found a moderate positive correlation among all GSEA pathways queried, supporting that global transcriptome patterns of *ETV4* and *ETV5* loss-of-function mutations are more similar to LY2874455 treated cells than dissimilar (Fig. 6D). These results in sum suggest that LY2874455 acts in part through inhibition of MAPK and PI3K-Akt signaling pathways in SCLC cells in vitro.

### In vivo efficacy of LY2874455 as a single agent and in combination with standard-of-care chemotherapy for treatment of SCLC

**Fig. 7:**
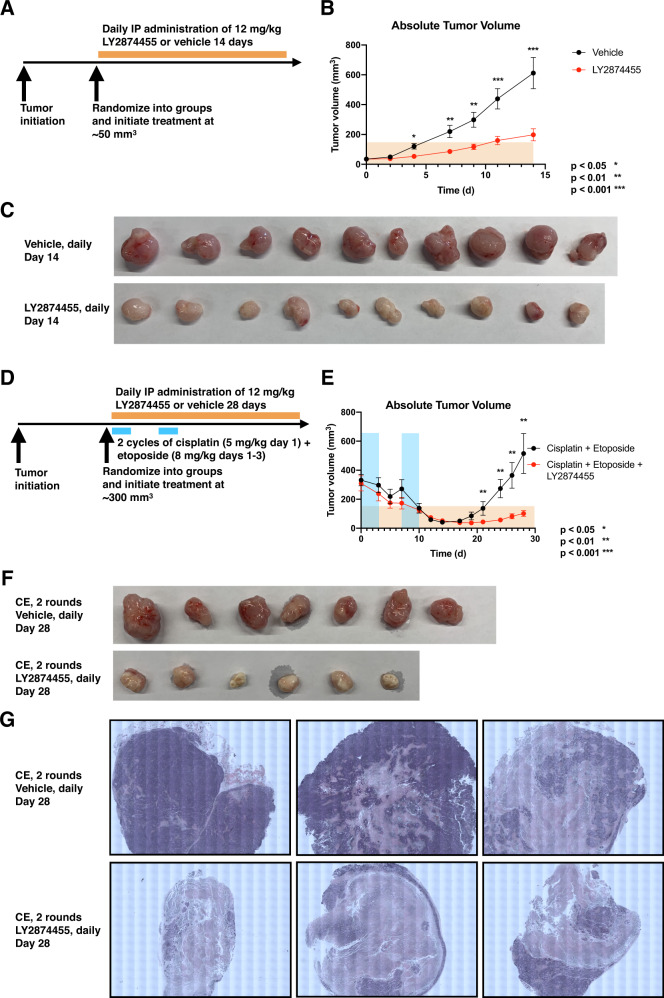
In vivo efficacy of LY2874455 as a single agent and in combination with standard-of-care for treatment of SCLC. **A** Experimental scheme for in vivo evaluation of single agent LY2874455 in H526 xenograft model. **B** H526 xenograft tumor growth comparing daily intraperitoneal administration of 12 mg/kg LY2874455 (*n* = 10) compared to vehicle (*n* = 10). Error bars represent standard error of the mean. **C** Representative photograph of tumors at 14 days of treatment. **D** Experimental scheme for evaluation of LY2874455 in combination with cisplatin and etoposide. **E** H526 xenograft tumor growth comparing daily intraperitoneal administration of 12 mg/kg LY2874455 in combination with cisplatin and etoposide (*n* = 6) compared to only cisplatin and etoposide (*n* = 7). Error bars represent standard error of the mean. **F** Representative photograph of tumors at 28 days of treatment. CE = cisplatin and etoposide. **G** Representative images of hematoxylin and eosin stained sections.

Finally, we sought to determine the in vivo efficacy of LY2874455 as a potential therapy for SCLC. We first evaluated LY2874455 as a single agent against human SCLC xenografts established in immunocompromised mice (Fig. 7A). We found a statistically significant reduction in tumor growth rate and final volume in the experimental group at the end of a 14-day treatment period when LY2874455 was administered daily at 12 mg/kg intraperitoneally (Fig. 7B). The mice in the experimental arm tolerated treatment with <10% reductions in weight compared to vehicle control. We then evaluated efficacy of LY2874455 when administered in combination with standard-of-care chemotherapy. Upon reaching enrollment volume, mice were randomized into either a group receiving two cycles of cisplatin and etoposide with previously established dosing parameters [8], or a group receiving the two cycles of combination chemotherapy alongside daily intraperitoneal administration of 12 mg/kg LY2874455 (Fig. 7E). We found that while the control group experienced an estimated 90% initial reduction in tumor volume following two cycles of combination chemotherapy, the mice quickly relapsed. The experimental group experienced a similar 90% reduction in tumor volume, as well as a prolonged suppression of tumor volume attributable to LY2874455, consistent across two models (Fig. 7F, Supplementary Fig. 7A). We compared the growth rates of tumors treated with single agent LY2874455 with the regrowth rates of tumors treated with combination chemotherapy with LY2874455. In the single LY2874455 treated group, the average daily rate of growth over 2 weeks is estimated at 11.65 mm^3^/day while in the triple combination group, the average daily rate of growth over 2 weeks is estimated to be 3.55 mm^3^/day. This suggests that regrowth in the triple combination group may be slowed compared to the single agent group. Following day 28 of treatment, we harvested the tumors for histological analysis. By hematoxylin and eosin staining, we noted striking areas of tumor necrosis in the LY2874455 treated group compared to the control (Fig. 7G), further supporting efficacy of LY2874455 in combination with cisplatin and etoposide for control of surviving tumor fractions.

## Discussion

Here, we present of a framework for deriving molecular insight into cancer recurrence in a time-dependent manner, leveraging both characterization of the intermediate transition to persistence state and unbiased global transcriptomics. This model was designed to test developing regulatory programs in the context of SCLC, a highly recalcitrant disease with rapid development of chemoresistance after combination chemotherapy. While mechanisms of resistance have been explored in the context of this disease, to our knowledge no study has performed unbiased global analysis on SCLC cells in a time-dependent fashion. Through this approach, we demonstrate the presence of a unique transcriptional state in SCLC DTP clones that is transient. This finding facilitated the identification of signaling pathways that regulate transcription factors which we show to be important for cell persistence. Other studies have also used a developmental framework in the context of resistance to uncover previously unappreciated molecular states and vulnerabilities [18, 19]. With the rapid decrease in sequencing costs and the miniscule amount of input RNA required, such an approach can be successfully applied to the study of small numbers of DTPs across cancer types and models. Extending this approach to directly study the response of human SCLC to therapy using circulating tumor cells collected longitudinally is likely to uncover molecular resistance mechanisms and allow the design of follow up therapeutic strategies. Given the highly efficacious cytoreduction seen across a majority of SCLC patients in response to combination chemotherapy, we argue that a combinatorial approach with combination chemotherapy and specific targeting against residual disease in SCLC may be a successful avenue towards improving survival in this aggressive disease.

Our gene ontological comparisons against embryonic diapause systems demonstrate a number of similar biological processes between the transient SCLC DTP state and embryonic diapause. Recently, similar observations have been made across a variety of malignancies, including colon cancer, malignant melanoma, and acute myeloid leukemia [33–35]. Such a state has been shown to be marked by down-regulation of MYC activity [23], which is thought to promote a cellular dormancy facilitating survival in response to chemotherapy. Such accumulating evidence serves as strong impetus to further uncover specific molecular pathways underlying the transitions to and from this embryonic diapause state. The underlying mechanisms may lend themselves to the development of novel classes of therapeutics that could work in conjunction with cytoreductive chemotherapies to produce stable remissions.

Our studies on the PEA3 transcription factors ETV4 and ETV5 uncover a previously unrecognized role for these transcription factors in human SCLC biology. The effect of *ETV4* and *ETV5* knockdown on SCLC clonogenic growth capacity following cisplatin and etoposide challenge underscore the value of a time course-based approach and implicate these genes in chemoresistance. Interestingly, we appreciated an additive effect of concomitant *ETV4* and *ETV5* knockdown in clonogenic regrowth, suggesting overlapping function between these two genes in this context. Notably, a previous study in mouse models of SCLC had uncovered a role for the mouse homolog of human *ETV4* in promoting metastatic dissemination [16]. ETV4 has also been previously implicated in regulating metastasis in non-small cell lung cancer through transcriptional control of extracellular matrix modifying enzymes [36, 37]. The clinical phenomena of drug resistance and metastasis are closely associated, but underlying molecular details linking the two phenomena have been lacking. ETV4 and ETV5 may serve as molecular members in common pathways underlying both chemoresistance and metastasis. Our studies raise further questions as to how *ETV4* and *ETV5* may be mediating post-chemotherapy regrowth in SCLC cells. Recent studies have demonstrated a role for another ETS-domain transcription factor ERG in promoting cellular growth in spite of extensive DNA damage [38, 39]. Additionally, there have been a number of studies that have implicated ETV4 in driving cell cycle progression [40–42]. Whether ETV4 and ETV5 serve as modulators of DNA damage signaling or as drivers of cell cycle progression in SCLC remains an open question for future study.

Our investigation of FGFR inhibitors for blocking downstream ETV4 and ETV5 activity led to the identification of LY2874455 as an efficacious agent, both for single use and in combination with cisplatin and etoposide. LY2874455 has been previously evaluated in a phase I clinical trial and was found to be well tolerated in patients with advanced solid tumors [43]. Unlike other forms of lung cancer, SCLC has demonstrated a relative dearth of targetable kinase driver mutations [2]. Thus, our work contributes to a growing body of literature identifying candidate kinase pathways and inhibitors in SCLC [4, 6, 7]. Compared to other FGFR inhibitors with broad inhibitory activity against multiple members of the FGFR group of receptors, we found LY2874455 to be uniquely efficacious in inhibition of FRS2 phosphorylation, a known signal transducing adaptor protein in intracellular FGFR signaling. Furthermore, we found evidence of downstream inhibition in both the PI3K-Akt and MAPK pathways known to be downstream of FGFR. We further found evidence of decreased ETV4 and ETV5 protein expression under LY2874455 treatment in SCLC cells. Lastly, our in vivo xenograft data demonstrating delayed tumor regrowth shows promise for further preclinical studies and clinical trials of LY2874455 with combination chemotherapy.

In conclusion, we demonstrate the utility of time course-based transcriptomic profiling in identifying transient cellular states and molecular targets associated with chemotherapy resistance and regrowth. We find evidence of a diapause-like state in SCLC DTPs following cisplatin and etoposide challenge paralleling other studies and lending support to the hypothesis that a common diapause-like signature underlies persistence in a subset of cancer cells across subtypes and treatment modalities. We demonstrate transience of the diapause-like state of DTP clones that wanes with increased cellular proliferation as DTPs expand out. We have further demonstrated the importance of both *ETV4* and *ETV5* expression in promoting efficient clonogenic regrowth in SCLC and identify the kinase inhibitor LY2874455 as a unique pan-FGFR inhibitor that blocks downstream MAPK and PI3K-Akt signaling in SCLC and demonstrates efficacy in curbing SCLC regrowth after cisplatin and etoposide challenge. While there remains further work to elucidate mechanistic underpinnings of ETV4 and ETV5 in this context and to fully define the kinase inhibition landscape of LY2874455, our study identifies molecular targets in SCLC relapse biology and nominates a therapeutic candidate that could contribute to increased survival for patients with this aggressive malignancy.

## Materials and methods

### Cell culture

The cell lines H82 (CRL-5811), H209 (HTB-172), H524 (CRL-5831), H526 (CRL-5811), H1417 (CRL-5869), and H1963 (CRL-5982) were purchased from the American Type Culture Collection. Cell line identity was confirmed via short tandem repeat profiling. Cells were maintained in RPMI 1640 (Thermo Fisher, 11875093) supplemented with 10% by volume heat inactivated fetal bovine serum (HI FBS) (Thermo Fisher, 10082147) and primocin (InvivoGen, ant-pm-2) at a final concentration of 50 μg/mL, hereafter referred to as standard RPMI. All cell lines were maintained at 37 °C in humidified chambers with 5% CO_2_. All cell lines were used for no longer than 20 passages.

### Antibodies and reagents

The following reagents were used in cell culture experiments: cisplatin (Tocris, 2251), etoposide (Millipore Sigma, E1383), LY2874455 (Selleckchem, S7057), infigratinib (Selleckchem, S2183), erdafitinib (Selleckchem, 8401), cabozantinib malate (Selleckchem, S4001), and lucitanib (MedChemExpress, HY-15391). Cisplatin was dissolved in normal saline for stock preparations. All other small molecules were dissolved in DMSO for stock preparations. The following antibodies were used for immunoblotting experiments: α-p-FRS2 antibody (Cell Signaling Technology, 3864 S), α-p-Erk1/2 (Cell Signaling Technology, 9101 S), α-Erk1/2 (Cell Signaling Technology, 9102 S), α-p-Akt (S473) (Cell Signaling Technology, 9271 S), α-Akt (Cell Signaling Technology, 9272 S), α-ETV4 (Abcam, ab189826), and α-ETV5 (Thermo Fisher, PA5-66555).

### Cell viability assay

For cell viability assays, starting cell densities were adjusted based on time points. For 72 h time points, 3 × 10^3^ to 5 × 10^3^ cells were seeded in 50 μL per well in a white, flat-bottom 96-well plate. For later time points (7 day and 14 day), 200–500 cells were seeded in 50 μL per well. After seeding, cells were incubated for 12 h prior to starting any drug exposure. For single agent titrations, 2x stocks of each dilution were prepared in standard RPMI and 50 μL of each was added to each well in either triplicate or quadruplicate. For dual agent titrations, 4x stocks of each dilution for each agent were prepared in standard RPMI and 25 μL of each agent was added to each well in triplicate. Upon reaching endpoint, CellTiter-Glo viability reagent (Promega, G7570) was used according to manufacturer’s instructions for viability determination.

### Flow cytometry

For flow cytometry assays, cells sampled at each time point were first centrifuged at 200 rcf at 4 °C for 5 min. Supernatant was decanted and cells were washed with ice-cold PBS prior to being suspended in 500 μL of FACS buffer (2% HI FBS in PBS). For viable cell quantitation, 20 μL of counting beads (Thermo Fisher, C36950) were added to each sample. Prior to data acquisition, 25 μL 7-amino-actinomycin D (Thermo Fisher, 00-6993) was added per sample to allow for viable cell detection. Samples were acquired on a BD LSRFortessa and data analysis was performed using FlowJo (Version 8.8.7). The same gating strategy was applied to all time points analyzed and total viable cell number was determined using normalization to counting beads.

### RNA sequencing and analysis

Cell samples were collected at indicated time points and kept on ice. Each sample was washed thrice with ice-cold PBS. Samples were then submitted to the Technology Center for Genomics and Bioinformatics (TCGB) for RNA isolation, rRNA depletion, cDNA library construction, indexing, and sequencing. Samples were pooled by cell line and sequenced on a single lane of HiSeq 3000 to generate 25–30 million 50-bp single reads per sample. Raw and processed sequencing data generated in this study have been deposited in the NCBI Gene Expression Omnibus (GEO) repository under accession number GSE214342. Following sequencing, individual fastq files were aligned to reference genome (hg38) using HISAT2 and counts were enumerated using HTSeq. All data analysis was implemented with R (Version 4.0.3). The following packages were used for analysis: *DESeq2*, *edgeR*, and *fgsea* [44, 45]. For all differential gene expression analyses, we used adjusted *p* < 0.05 as a statistical significance threshold. For diapause gene signature scoring, we utilized the top differentially expressed genes in comparison of murine diapause embryos and E4.5 epiblast with adjusted *p* < 0.05. Mouse genes were converted to human homologs and geometric means were calculated across time points for each dataset and normalized to a mean of 0 and standard deviation of 1.

### Immunoblot

For immunoblot analyses, all samples were kept on ice and washed thrice with ice-cold PBS. Samples were resuspended in appropriate volumes of 1X cell lysis buffer (Cell Signaling Technology, 9803 S) supplemented with Halt protease inhibitor cocktail (Thermo Fisher, 78430) and Simple Stop 2 phosphatase inhibitor cocktail (Gold Bio, GB-451-1). Suspensions were incubated at 4 °C with constant agitation for 30 min and then spun down at 16,000 rcf in a bench top centrifuge. Supernatants were collected into fresh tubes and protein concentration was determined by bicinchoninic acid assay (Thermo Fisher, 23225) according to manufacturer’s instructions. Lysates were brought to a concentration of either 0.5 or 1 μg/μL with 1x Laemmli buffer (Bio-rad, 1610747) and 1.25% v/v 2-mercaptoethanol (Millipore Sigma, M6250). Lysates were incubated at 95 °C for 5 min followed by at 4 °C for 5 min prior to gel electrophoresis. Briefly, lysates were run on stain-free 4–15% gradient polyacrylamide gels that allow for total protein quantification (Bio-rad, 4568085) for 1 h at 120 V. 10 μL of protein ladder (Bio-rad, 1610373) was used for size determination in each gel. Following gel electrophoresis, protein stain was activated with 5 min of UV exposure and imaged. Protein was transferred to 0.45 μm nitrocellulose membranes (Bio-rad, 1620235). Membranes were blocked with 5% w/v bovine serum albumin dissolved in Tris-buffered saline with 0.1% v/v Tween 20 (Millipore Sigma, P1379) (TBST) for 1 h at 23 °C. Membranes were incubated with primary antibodies diluted in blocking buffer overnight at 4 °C on a rocker. The following day, membranes were washed thrice with TBST for 10 min on a rocker at 23 °C and incubated in the appropriate secondary antibody-horseradish peroxidase conjugate for 1 h on a rocker at 23 °C. Membranes were then washed thrice with TBST for 10 min on a rocker at 23 °C. Membranes were then incubated in chemiluminescent substrate (Thermo Fisher, 34095) for 5 min at 23 °C prior to imaging on a ChemiDoc MP Imaging System.

### Lentiviral transduction

Single cell suspensions were prepared by dissociating cells in Accumax (Millipore Sigma, A7089) at 23 °C for 5 min. Enzymatic digestion was neutralized with an equivalent volume of complete culture medium. Cells were pelleted by centrifugation at 500 rcf and resuspended in an appropriate volume of culture medium without antibiotics, counted, and cultured overnight. The following day, cells were seeded onto a 96-well plate at a density of 1 × 10^6^ cell/mL and polybrene (Millipore Sigma, TR-1003-G) was added to a final concentration of 5 μg/mL. Lentiviral supernatants were added and cells were incubated for 24 h. The following lentiviruses and multiplicity of infection (MOI) were used for stable line generation: lentiCas9-blast (MOI: 0.7, Addgene, 52962-LV), ETV4 shRNA (MOI: 10, GeneCopoeia, LPP-HSE053982-LVE002-a-050), ETV5 shRNA (MOI: 10, GeneCopoeia, LPP-HSE095597-LVE001-a-050), Scramble-eGFP (MOI: 10, GeneCopoeia, LPP-CSECTR001-LVE001-025), Scramble-mCherry (MOI:10, GeneCopoeia, LPP-CSECTR001-LVE002-025), ETV4-N-Flag (MOI:5, GeneCopoeia, LPP-I1227-Lv102-050), ETV5-N-3XHA (MOI: 5, GeneCopoeia, LPP-F0800-Lv118-050), and eGFP (MOI: 5, GeneCopoeia, LPP-mEGFP-Lv105-100-C). Cells were further expanded for 48 h prior to selection with the appropriate antibiotics. Antibiotic selection was performed at the following final concentrations: blasticidin (10 μg/mL), puromycin (1 μg/mL), geneticin (800 μg/mL). Following 1 week of selection, cells were maintained in selection media at the following concentrations: blasticidin (5 μg/mL), puromycin (500 ng/mL), geneticin (400 μg/mL).

### Gene expression quantification by polymerase chain reaction

Total RNA was isolated via commercially available kit according to manufacturer’s instructions (Qiagen, 74004). Up to 1 ug of RNA was used as template for cDNA synthesis according to manufacturer’s instructions (Qiagen, 1708891). Commercially sourced TaqMan assay probes were used for real time polymerase chain reaction in TaqMan Fast Universal Master Mix, no AmpErase UNG (Thermo Fisher, 4352042). The following probes were used: ETV4 (Thermo Fisher, 4448892, Hs00383361_g1), ETV5 (Thermo Fisher, 4448892, Hs00927557_m1), and β-actin (Thermo Fisher, 4326315E). Relative gene expression changes between control and experimental samples were determined using the following formula: 2^-ΔΔ*Ct*^, where Δ*Ct* is the difference in *Ct* between the gene of interest and housekeeping gene and ΔΔ*Ct* is the difference in Δ*Ct* between the experimental and control groups.

### Clonogenic recovery assay

Cells were single cell dissociated and resuspended in a suspension of 1% methylcellulose (R&D Systems, HSC001) in standard RPMI to a final density of 5 × 10^4^ cells/mL. 1 × 10^5^ cells were seeded into 12-well plates in at least triplicate per condition. Unless otherwise stated, each assay was repeated three times. Colony counting was performed under light microscopy at indicated time points.

### CRISPR-Cas9 mutant generation

Design tools provided by Synthego and Benchling were used for gRNA spacer sequence design. Sequences with maximized off-target scores were prioritized. For each gene target, four spacer sequences were chosen for synthesis into a gRNA expression plasmid (Addgene, 41824). Spacer sequence cloning was performed as previously described [46]. Briefly, spacer sequences were synthesized with 40 bp overlap with the expression vector. Vector was linearized via incubation with restriction enzyme AflII (New England BioLabs, R0520S) according to manufacturer’s instructions. Spacer sequence was inserted into expression plasmid via Gibson assembly (New England BioLabs, E2611S) according to manufacturer’s instructions. Gibson assembly reaction mixtures were used to transform chemically competent *E. coli* (Thermo Fisher, C737303) and plasmids were purified using commercially obtained plasmid DNA purification kits (Qiagen, 12362). Successful spacer sequence insertion was verified by Sanger sequencing. Established lines with stable *S. pyogenes* Cas9 expression were transfected with gRNA expression plasmids using a 4D-Nucleofector (Lonza, AAF-1002B), X-unit (Lonza, AAF-1002X), and SF cell line electroporation reagent kit (Lonza, V4XC-2032). For electroporation, cells were removed from blasticidin selection media and allowed to incubate overnight in standard RPMI prior to electroporation. For 20 μL reactions, 600,000 cells and 1 or 2 μg of purified plasmid were used per reaction. Protocol DN-100 was empirically found to yield the greatest transfection efficiency with minimal cell death and was used for every electroporation. Sanger sequencing of target loci was performed one week following electroporation to determine gRNA spacer sequences with the greatest cutting efficiency. Lines transfected with the best cutting gRNA spacer sequences were expanded, single cell dissociated, and seeded at a density of 500 cells per cm^2^ in single 10-cm dishes in a suspension of 1% methylcellulose in standard RPMI. Individual clones were allowed to expand out to colonies of 50-100 cells over two weeks. Monoclonal colonies were then picked and seeded into individual wells of a 96 well plate for further expansion. Sanger sequencing was used to identify clones with homozygous frameshift mutations for further study.

### In vivo xenografts

For xenograft establishment, female NOD *scid* gamma (NSG) mice (The Jackson Laboratory, 005557) 6-8 weeks of age were shaved. Minimum sample size per group was determined using a type I error rate of 0.05, power of 0.80, and the ability to detect a 50% reduction in tumor volume in each experimental group. Cells were dissociated as previously described and were resuspended in a mixture of standard RPMI and Matrigel (Corning, 354234) prepared at a 1:1 ratio at a density of 1 × 10^7^ cells/mL. 100 μL of cell suspension was injected subcutaneously into the right flanks of each mouse under isoflurane (Henry Schein, G125F19A). Mice were randomized to control or experimental groups at time of tumor initiation. Mice were monitored daily for tumor growth. Tumor lengths and widths were measured twice every week and volumes were estimated using the following formula: ((length)(width)^2^)/2. Mouse weights were also monitored twice weekly. Xenografts were only established on the right flanks of each mouse. Enrollment volumes were 50 mm^3^ for single agent LY2874455 evaluation and 300 mm^3^ for LY2874455 combination with standard-of-care evaluation. Cisplatin and etoposide combination chemotherapy was administered in weekly rounds consisting of a single 5 mg/kg dose of cisplatin administered intraperitoneally on day 1 and daily 8 mg/kg doses of etoposide administered intraperitoneally on days 1, 2, and 3. LY2874455 was administered intraperitoneally daily over each indicated time course. Experimental and control treatments were administered in an unblinded fashion. Cisplatin was dissolved in normal saline while etoposide and LY2874455 were prepared in solutions of 5% v/v DMSO (Millipore Sigma, D2650), 5% v/v Tween 80 (Millipore Sigma, P4780), 30% v/v polyethylene glycol 300 (Millipore Sigma, 91462), and 50% v/v H_2_O (Corning, 25055CV). On the days of chemotherapy administration, mice were also given a subcutaneous bolus of 500 μL Lactated Ringer’s solution to mitigate potential cisplatin nephrotoxicity. All animal studies were performed with compliance to ethical regulations and with approval from IUCAC.

### Histology

Upon reaching endpoints, xenograft tumor samples were dissected and fixed in 4% formaldehyde overnight at 23 °C. Samples were further incubated in a solution of 25% w/v sucrose dissolved in distilled H_2_O overnight at 4 °C. Samples were paraffin embedded, sectioned at 4 μm thickness, and stained with hematoxylin-eosin. Samples were imagined on a Zeiss Axio microscope.

### Immunofluorescence staining and imaging

Paraffin embedded sections were deparaffinized and antigen retrieval was performed in 10 mM sodium citrate buffer with 0.05% v/v Tween-20. Sections were then permeabilized in TBST with 0.1% v/v Triton X-100 (Millipore Sigma, X100) for 10 min at 23 °C. Samples were then washed briefly with TBST and blocked in Dako serum free protein block (Agilent, X090930-2). Primary antibodies were diluted according to manufacturer’s recommendations in protein block and samples were incubated overnight at 4 °C. The following day, samples were washed thrice with TBST for 10 min each at 23 °C and incubated with secondary antibodies diluted 500-fold in TBST for 1 h at 23 °C. Following another three TBST washes as previously described, samples were mounted (Vector Laboratories, H-1000-10) and imaged on a Zeiss Axio microscope.

### Statistical analysis

All statistical analysis was performed using Prism (Version 9.0.0). All statistical tests performed were two-sided. Mann–Whitney *U* tests were used for tumor volume comparisons.

### Study approval

All mouse studies were approved by the Institutional Animal Care and Use Committee at the University of California, Los Angeles under protocol ARC-2008-123.

## Supplementary information

Combined supplementary materials

## Data Availability

All relevant data are available by request. Raw and processed sequencing data generated in this study have been deposited in the NCBI Gene Expression Omnibus (GEO) repository under accession number GSE214342.
