# Supplementary material for: Targeting PEA3 transcription factors to mitigate small cell lung cancer progression
Source: Oncogene. 2022 Dec 13;42(6):434–48. doi: 10.1038/s41388-022-02558-6 (PMC9898033; doi:10.1038/s41388-022-02558-6)
Supplement: Supplementary file 1 — Combined supplementary materials [file 41388_2022_2558_MOESM1_ESM.pdf]

SUPPLEMENTAL INFORMATION

1. Supplemental figure legends
2. Supplemental figures
3. Table S1 – list of diapause DEGs

Figure S1

A) Additional cisplatin and etoposide dual titrations across an expanded panel of SCLC cell lines: H146, H209, H524, and H1417.
B) Additional EdU labeling across time points in response recovery time course in H526 and H1963 cell lines.
C) Gating strategy for the quantification of viable cells at each time point.
D) Representative flow cytometry scatterplots with 7-AAD signal plotted against forward scatter demonstrating viable population gate at various time points in H82.

Figure S2

A) Distance clustering separated by cell line demonstrating samples to be excluded in downstream analysis in each response-recovery time course.
B) Plots of principal components 1 and 2 for each cell line time course dataset.

Figure S3

A) Pearson correlation heatmap of expression of all ETS group transcription factors among 49 SCLC cell lines from the Cancer Cell Line Encyclopedia (CCLE).
B) Pearson correlation heatmap of expression of all ETS group transcription factors among 47 primary SCLC samples from George et al.
C) Dependencies across 25 SCLC lines on ETV4 and ETV5 RNAi perturbation, ASCL1 and NEUROD1 included as positive controls.
D) Dependencies across 19 SCLC lines on ETV4 and ETV5 CRISPR perturbation, ASCL1 and NEUROD1 included as positive controls.

Figure S4

A) Summary of frameshift mutations in four evaluated H526 sublines at ETV4 exon 5.
B) Summary of frameshift mutations in four evaluated H526 sublines at ETV5 exon 6.
C) Evaluation of off-target editing at ENO2 and WDR93.
D) Quantitative polymerase chain reaction of H82 and H526 lines stably transduced with shRNA targeting ETV4 and ETV5.
E) Quantitative polymerase chain reaction of H526 line stably transduced with cDNA for overexpression of ETV4 and ETV5.
F) Cellular growth curves of stably-transduced shRNA lines generated from H82 and H526 parental lines by CellTiter-Glo. Each time point was measured in quadruplicate.
G) Cellular growth curves of stably-transduced cDNA lines generated from H526 parental line by CellTiter-Glo. Each time point was measured in quadruplicate.

Figure S5

A) Summary of measured *in vitro* IC_{50} values for target binding of selected tyrosine kinase inhibitors.
B) Dual titrations of pan-FGFR inhibitors infigratininb and erdafitinib against lucitinib in H82 (left) and H526 (right).

Figure S6

A) Time course analysis of phosphorylation of Erk1/2 (T202/Y204) and Akt (S473) following exposure to 500 nM LY2874455 in H82 (left) and H526 (right).
B) Clonogenic regrowth assay in stably-transduced H526 lines expressing the following cDNA: GFP, ETV4, ETV5, ETV4 and ETV5. As above, cells were treated with 500 nM cisplatin and etoposide for 72 hours and then seeded in 1% methylcellulose prepared with a final concentration of 500 nM LY2874455 for quantification of clonogenic regrowth. Non-parametric t-test was used to determine statistical significance between groups.

Figure S7

A) H209 xenograft tumor growth comparing daily intraperitoneal administration of 12 mg/kg LY2874455 in combination with cisplatin and etoposide (n=5) compared to only cisplatin and etoposide (n=6). Error bars represent standard error of the mean.

Supplementary Figure 1

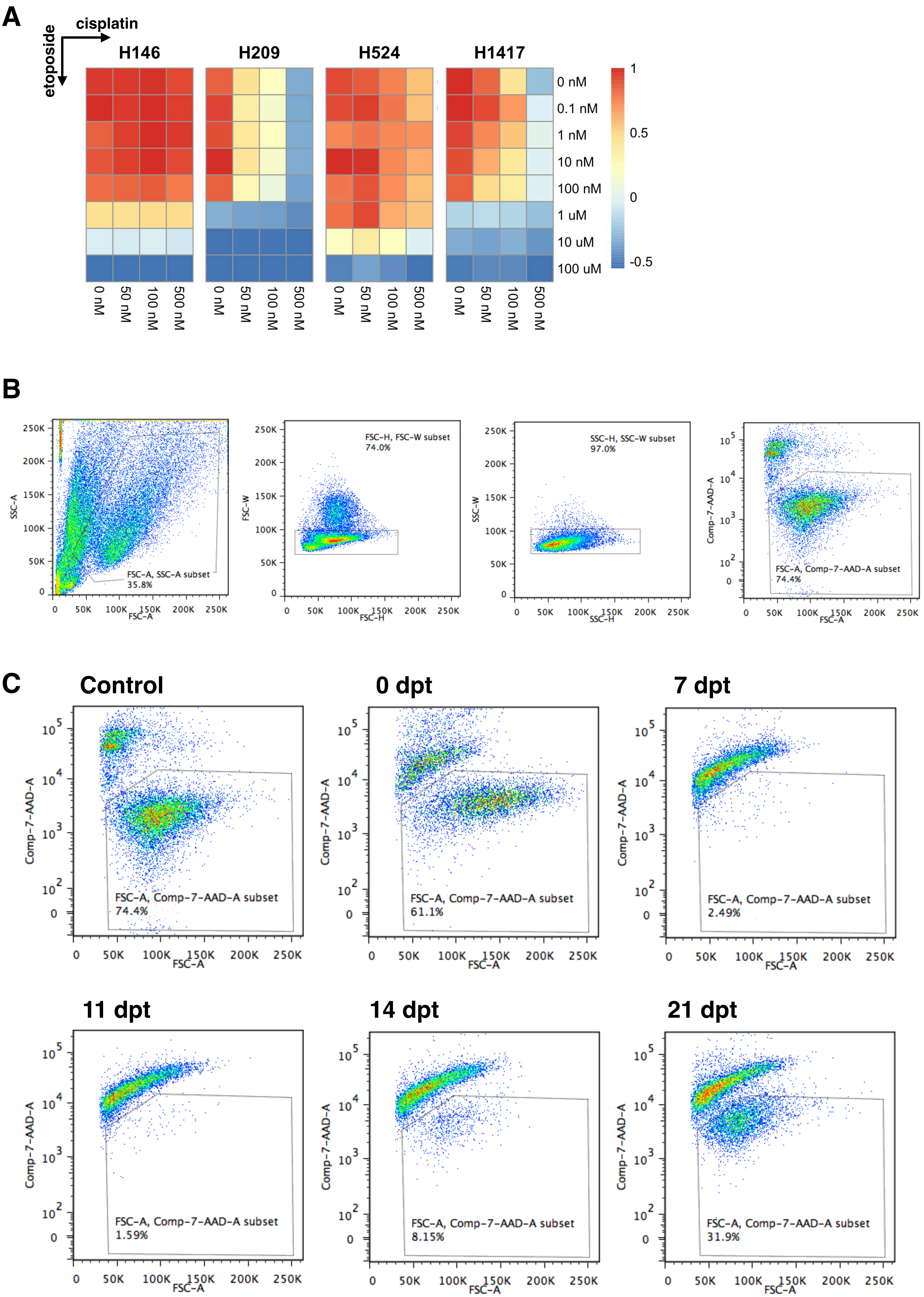

Supplementary Figure 1 (continued)

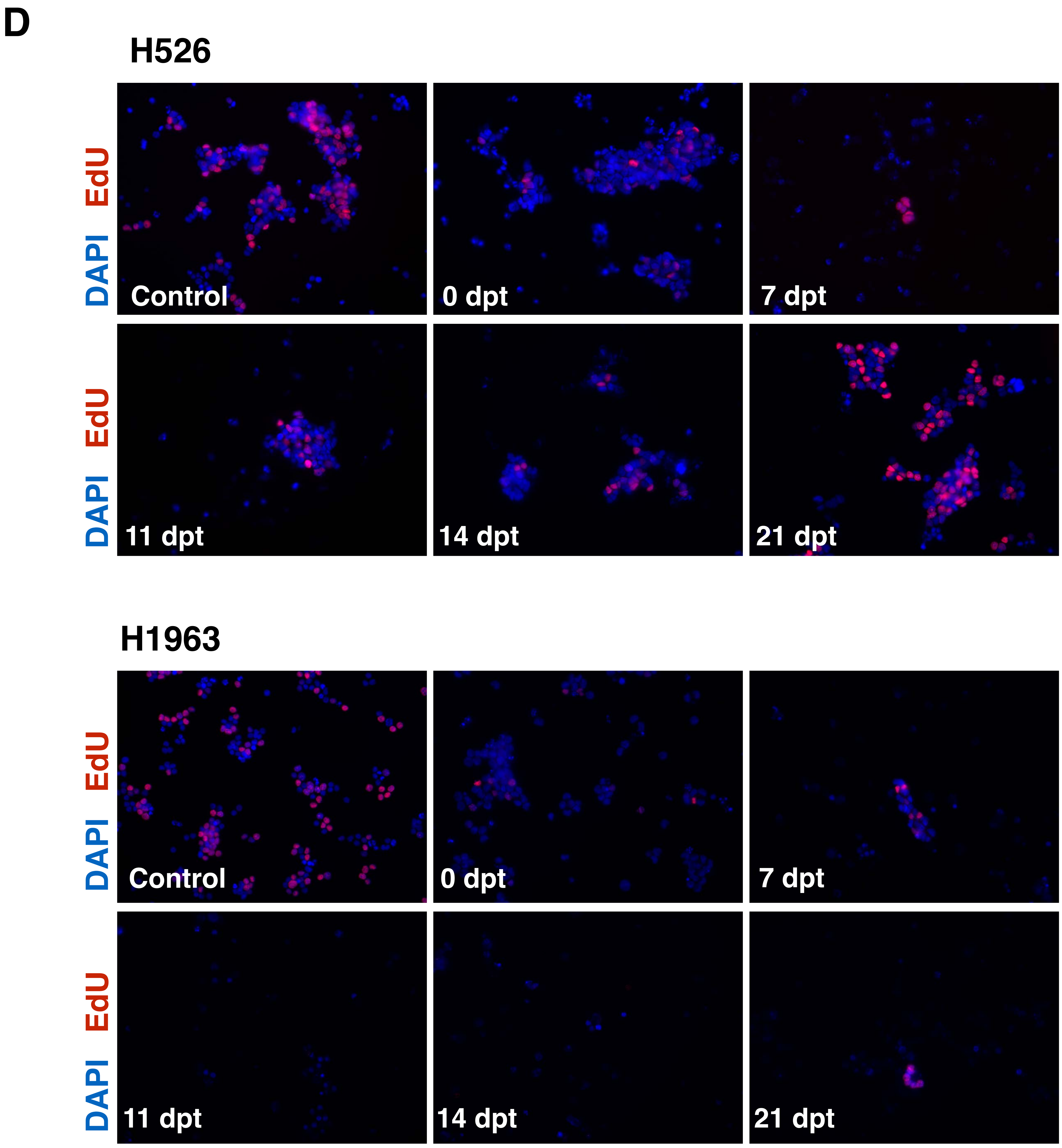

Supplementary Figure 2

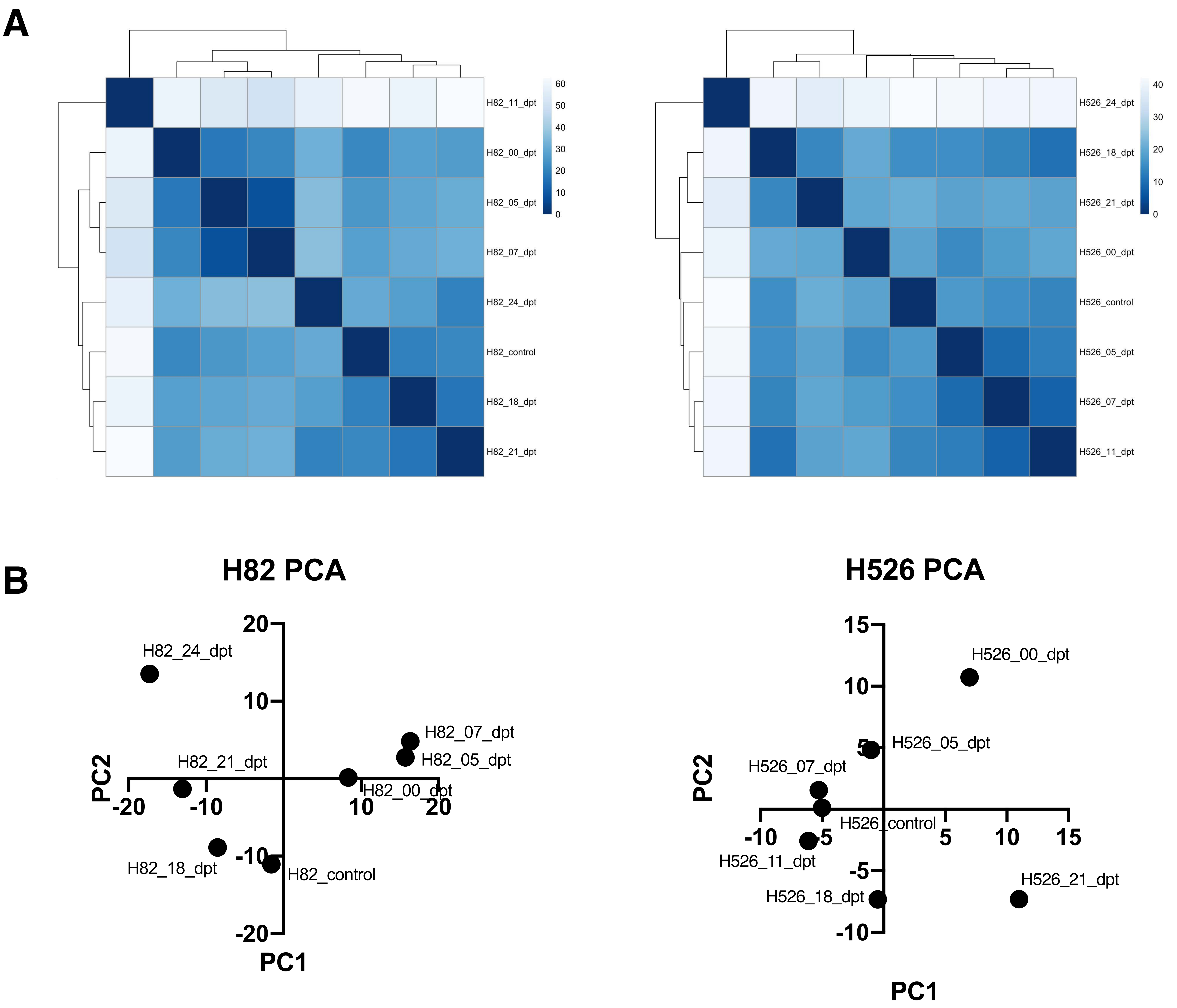

Supplementary Figure 3

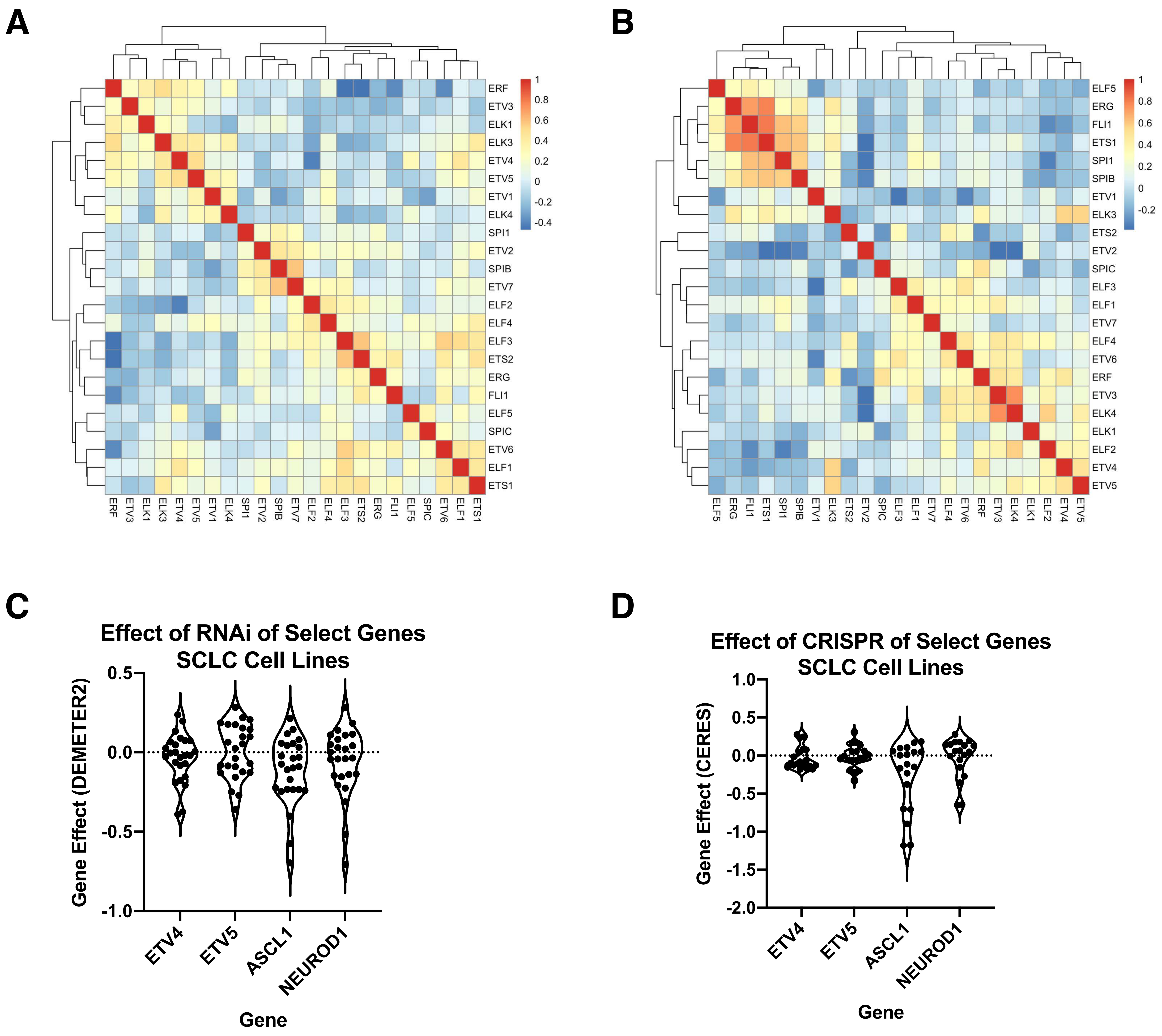

Supplementary Figure 4

A

SAMPLE	GUIDE TARGET	PAM SEQUENCE	INDEL %	MODEL FIT (R^2)	KNOCKOUT-SCORE
AC5	CTTTCCACAGCCCCACCACC	AGG	96	0.96	96
AD5	CTTTCCACAGCCCCACCACC	AGG	97	0.97	97
BD2	CTTTCCACAGCCCCACCACC	AGG	97	0.97	97
DC2	CTTTCCACAGCCCCACCACC	AGG	98	0.98	25

Cell Line

AC5

INDEL	CONTRIBUTION	SEQUENCE
-1	64%	TCTGGGGCTCCTTCTTGATCCTGGT ¦ -GTGGGGCTGTGGAAAGCTACTGTGGGGGTGGAGGGGACAGAGCAAGGCC
+1	32%	TCTGGGGCTCCTTCTTGATCCTGGT ¦ NGGTGGGGCTGTGGAAAGCTACTGTGGGGGTGGAGGGGACAGAGCAAGGC

AD5

INDEL	CONTRIBUTION	SEQUENCE
-1	97%	TCTGGGGCTCCTTCTTGATCCTGGT ¦ -GTGGGGCTGTGGAAAGCTACTGTGGGGGTGGAGGGGACAGAGCAAGGCC

BD2

INDEL	CONTRIBUTION	SEQUENCE
-1	47%	TCTGGGGCTCCTTCTTGATCCTGGT ¦ -GTGGGGCTGTGGAAAGCTACTGTGGGGGTGGAGGGGACAGAGCAAGGCC
+1	42%	TCTGGGGCTCCTTCTTGATCCTGGT ¦ NGGTGGGGCTGTGGAAAGCTACTGTGGGGGTGGAGGGGACAGAGCAAGGC
+2	8%	TCTGGGGCTCCTTCTTGATCCTGGT ¦ NNGGTGGGGCTGTGGAAAGCTACTGTGGGGGTGGAGGGGACAGAGCAAGG

DC2

INDEL	CONTRIBUTION	SEQUENCE
-3	73%	TCTGGGGCTCCTTCTTGATCCTGGT ¦ ---GGGGCTGTGGAAAGCTACTGTGGGGGTGGAGGGGACAGAGCAAGGCC
+1	25%	TCTGGGGCTCCTTCTTGATCCTGGT ¦ NGGTGGGGCTGTGGAAAGCTACTGTGGGGGTGGAGGGGACAGAGCAAGGC

B

SAMPLE	GUIDE TARGET	PAM SEQUENCE	INDEL %	MODEL FIT (R^2)	KNOCKOUT-SCORE
AC5	GCTACAAGACGACAGCTCAG	AGG	97	0.97	97
AD5	GCTACAAGACGACAGCTCAG	AGG	99	0.99	99
BD2	GCTACAAGACGACAGCTCAG	AGG	95	0.95	64
DC2	GCTACAAGACGACAGCTCAG	AGG	99	0.99	99

Cell Line

AC5

INDEL	CONTRIBUTION	SEQUENCE
+1	97%	TGCTCATGGCTACAAGACGACAGCT ¦ NCAGAGGAGGGGCTGTGCAGCTCCCGTTTGATCTTGGTTGGAGGTGGGGC

AD5

INDEL	CONTRIBUTION	SEQUENCE
+1	99%	TGCTCATGGCTACAAGACGACAGCT ¦ NCAGAGGAGGGGCTGTGCAGCTCCCGTTTGATCTTGGTTGGAGGTGGGGC

BD2

INDEL	CONTRIBUTION	SEQUENCE
+1	32%	TGCTCATGGCTACAAGACGACAGCT ¦ NCAGAGGAGGGGCTGTGCAGCTCCCGTTTGATCTTGGTTGGAGGTGGGGC
-3	24%	TGCTCATGGCTACAAGACGACA--- ¦ CAGAGGAGGGGCTGTGCAGCTCCCGTTTGATCTTGGTTGGAGGTGGGGCA
-2	18%	TGCTCATGGCTACAAGACGACAGC- ¦ -AGAGGAGGGGCTGTGCAGCTCCCGTTTGATCTTGGTTGGAGGTGGGGCA
-2	14%	TGCTCATGGCTACAAGACGACAGCT ¦ --GAGGAGGGGCTGTGCAGCTCCCGTTTGATCTTGGTTGGAGGTGGGGCA
-3	7%	TGCTCATGGCTACAAGACGACAGCT ¦ ---AGGAGGGGCTGTGCAGCTCCCGTTTGATCTTGGTTGGAGGTGGGGCA

DC2

INDEL	CONTRIBUTION	SEQUENCE
+1	99%	TGCTCATGGCTACAAGACGACAGCT ¦ NCAGAGGAGGGGCTGTGCAGCTCCCGTTTGATCTTGGTTGGAGGTGGGGC

C

ENO2

SAMPLE	GUIDE TARGET	PAM SEQUENCE	INDEL %	MODEL FIT (R^2)	KNOCKOUT-SCORE
AC5	CTGTGCACAGCCCCACCACC	AGG	0	1	0
AD5	CTGTGCACAGCCCCACCACC	AGG	0	1	0
BD2	CTGTGCACAGCCCCACCACC	AGG	0	1	0
DC2	CTGTGCACAGCCCCACCACC	AGG	0	1	0

WDR93

SAMPLE	GUIDE TARGET	PAM SEQUENCE	INDEL %	MODEL FIT (R^2)	KNOCKOUT-SCORE
AC5	CTTTCCCCCGCCCCCCCACC	AGG	0	1	0
AD5	CTTTCCCCCGCCCCCCCACC	AGG	0	1	0
BD2	CTTTCCCCCGCCCCCCCACC	AGG	0	1	0
DC2	CTTTCCCCCGCCCCCCCACC	AGG	0	1	0

Supplementary Figure 4 (continued)

D

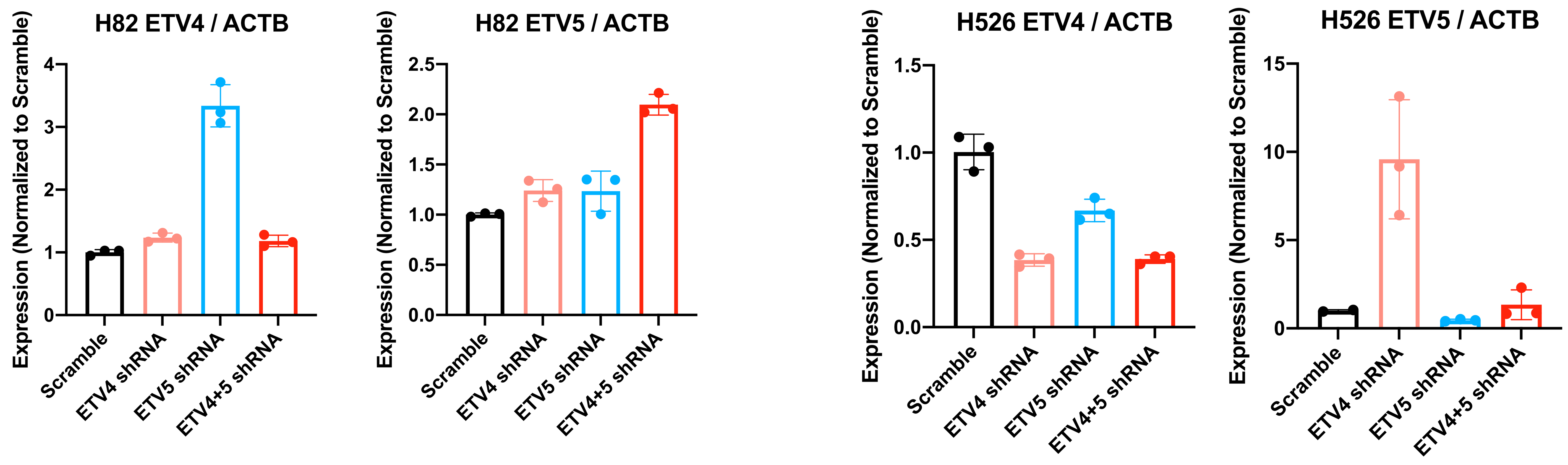

E

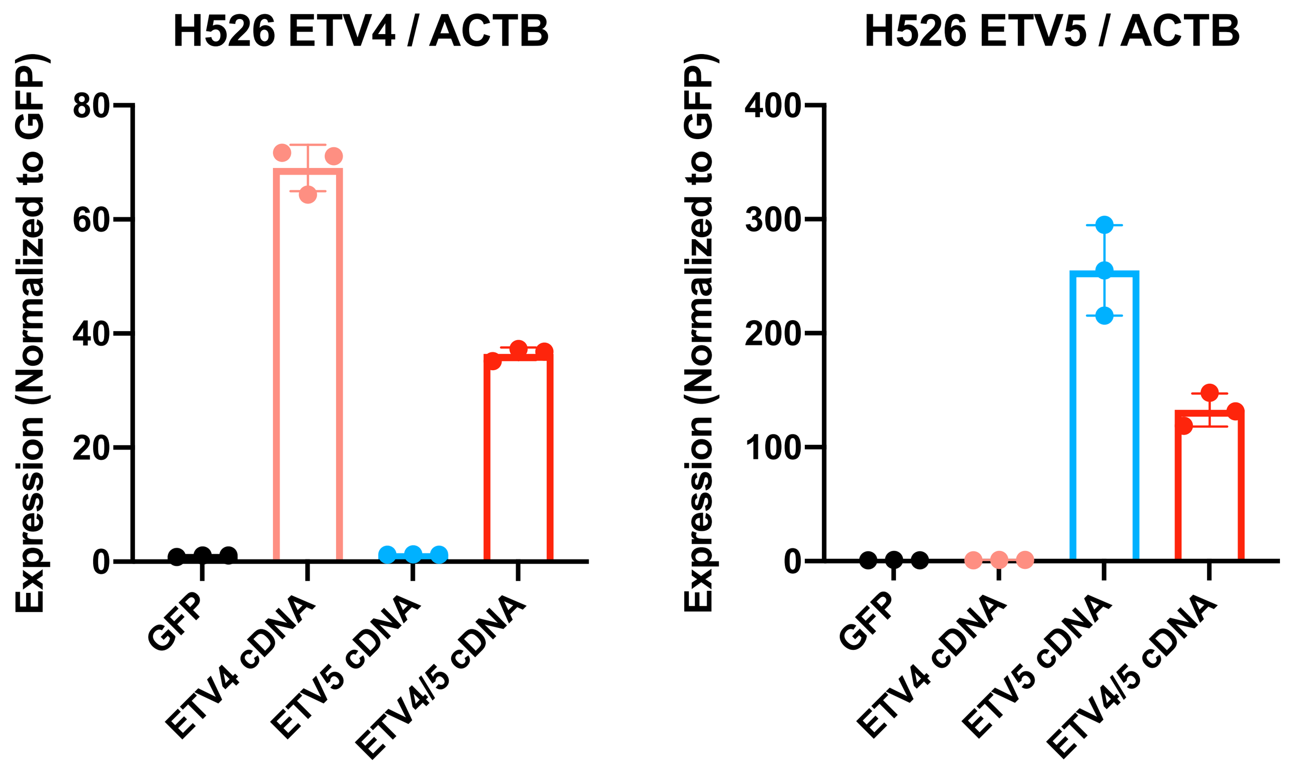

F

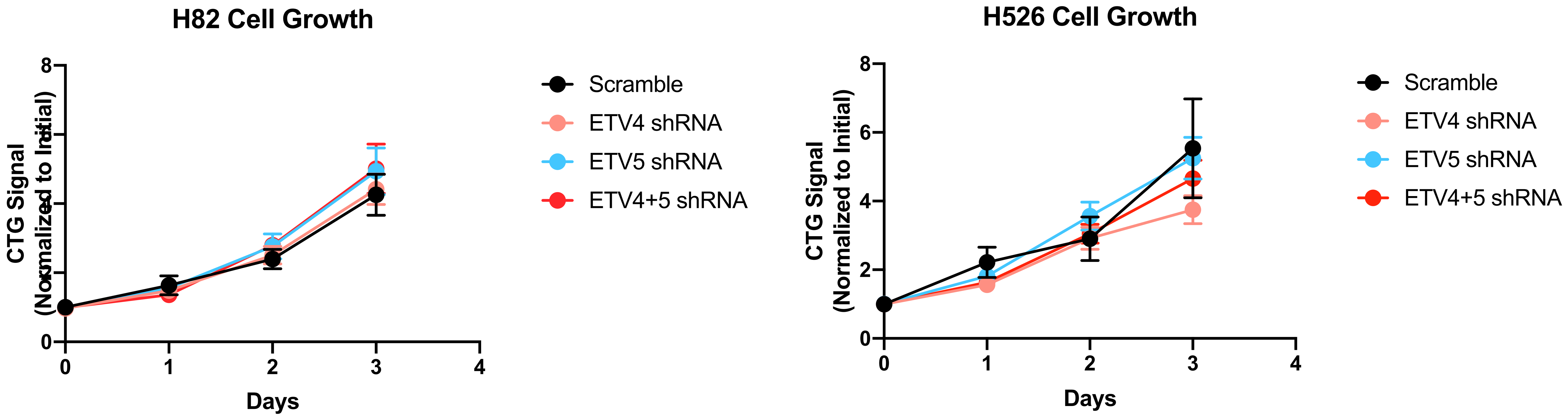

G

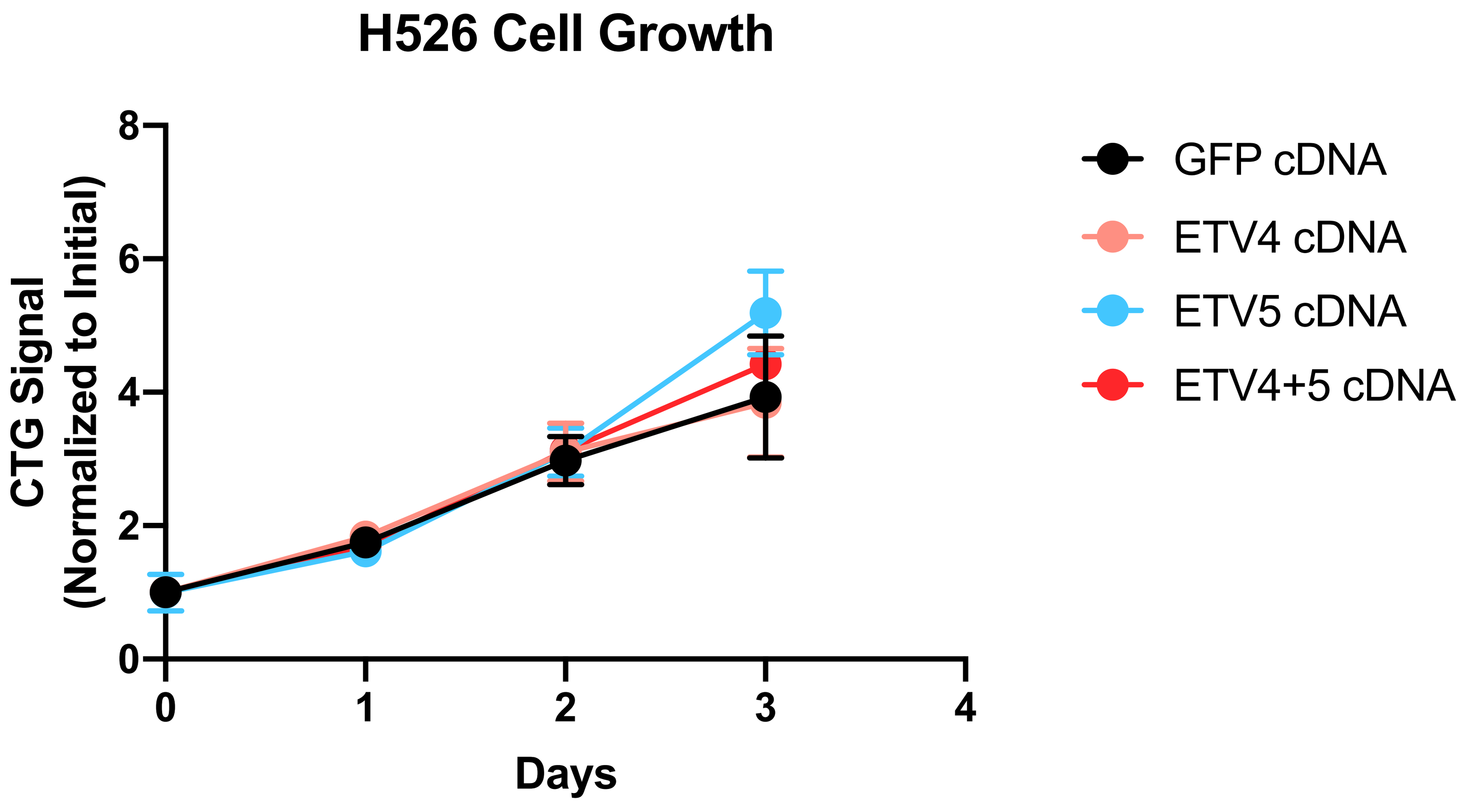

Supplementary Figure 5

A

Known IC50 for FGFR/VEGFR tyrosine kinase inhibitors (nM)

	FGFR1	FGFR2	FGFR3	FGFR4	VEGFR2	Notes
LY2874455	2.80	2.60	6.40	6.00	7.00	-
Infigratinib	0.90	1.40	1.00	-	-	40-fold selectivity for FGFR1/2/3 over FGFR4 and VEGFR2
Erdafitinib	1.20	2.50	3.00	5.70	36.80	20-fold selectivity for FGFR1/2/3/4 over VEGFRs
Roblitinib	-	-	-	1.10	-	1000-fold selectivity for FGFR4 over other FGFRs
Cabozantinib	-	-	-	-	0.035	-

B

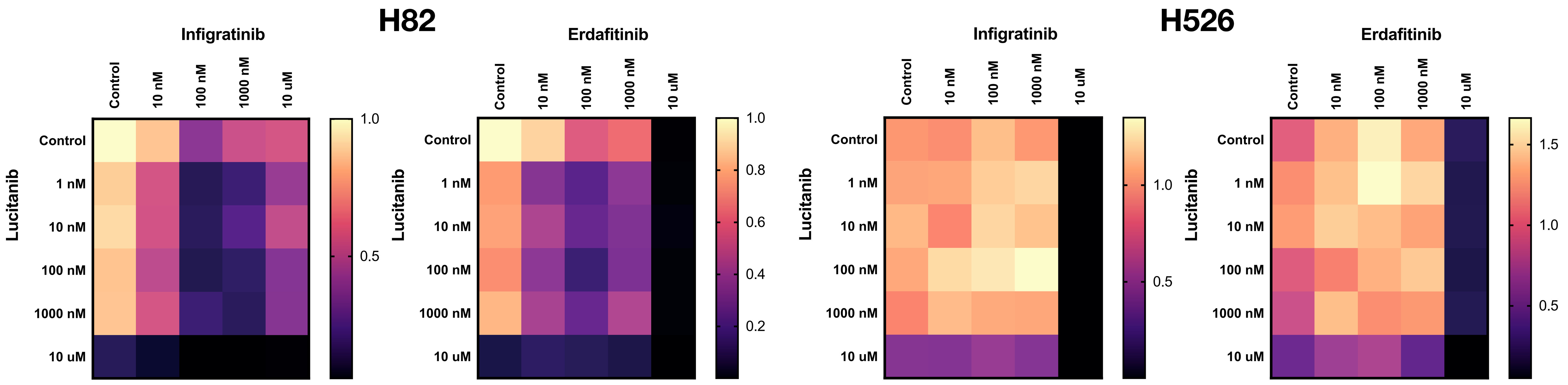

Supplementary Figure 6

A

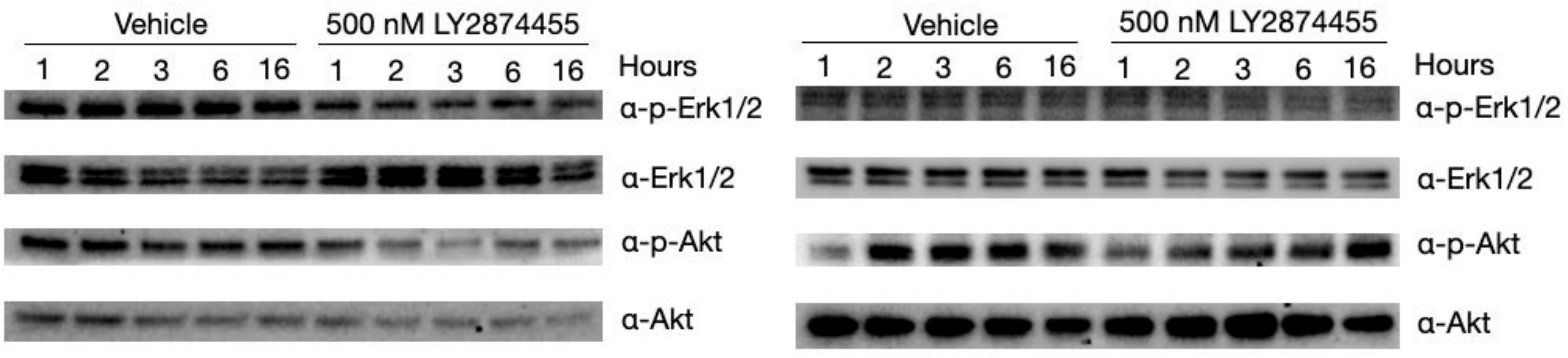

B

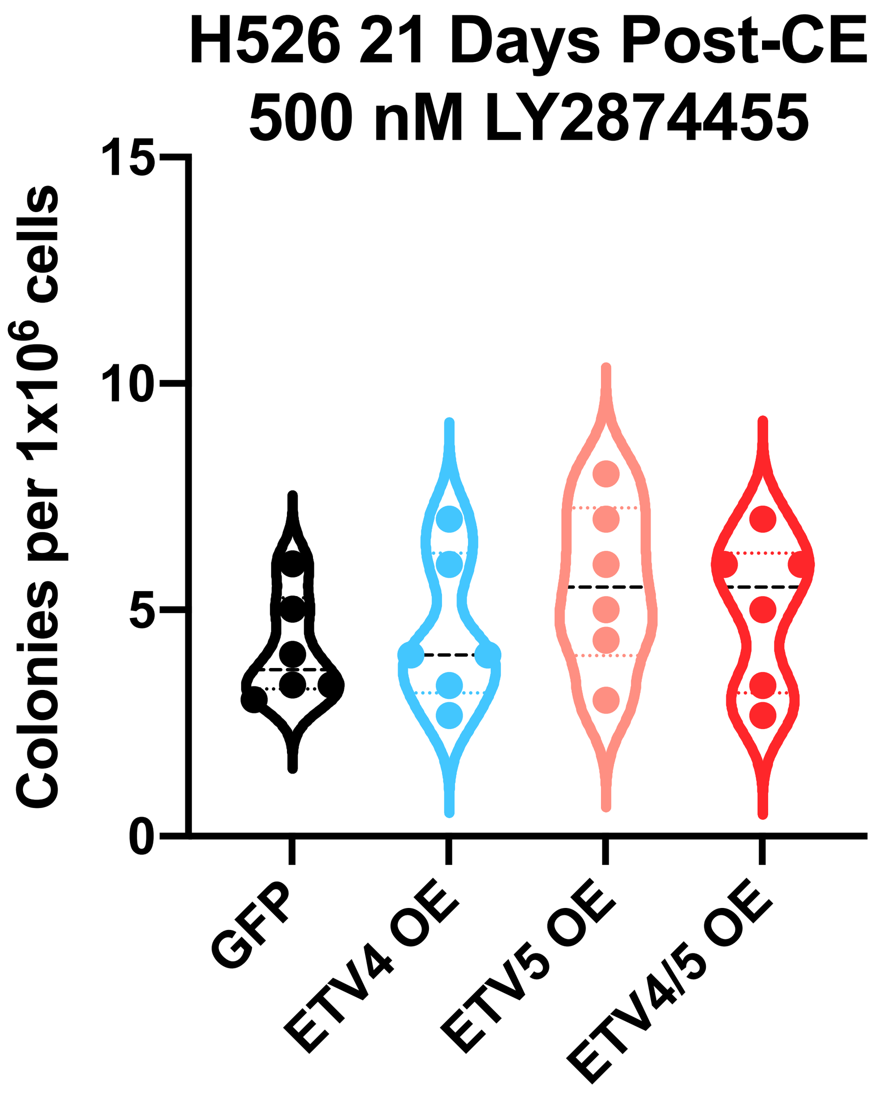

Supplementary Figure 7

A

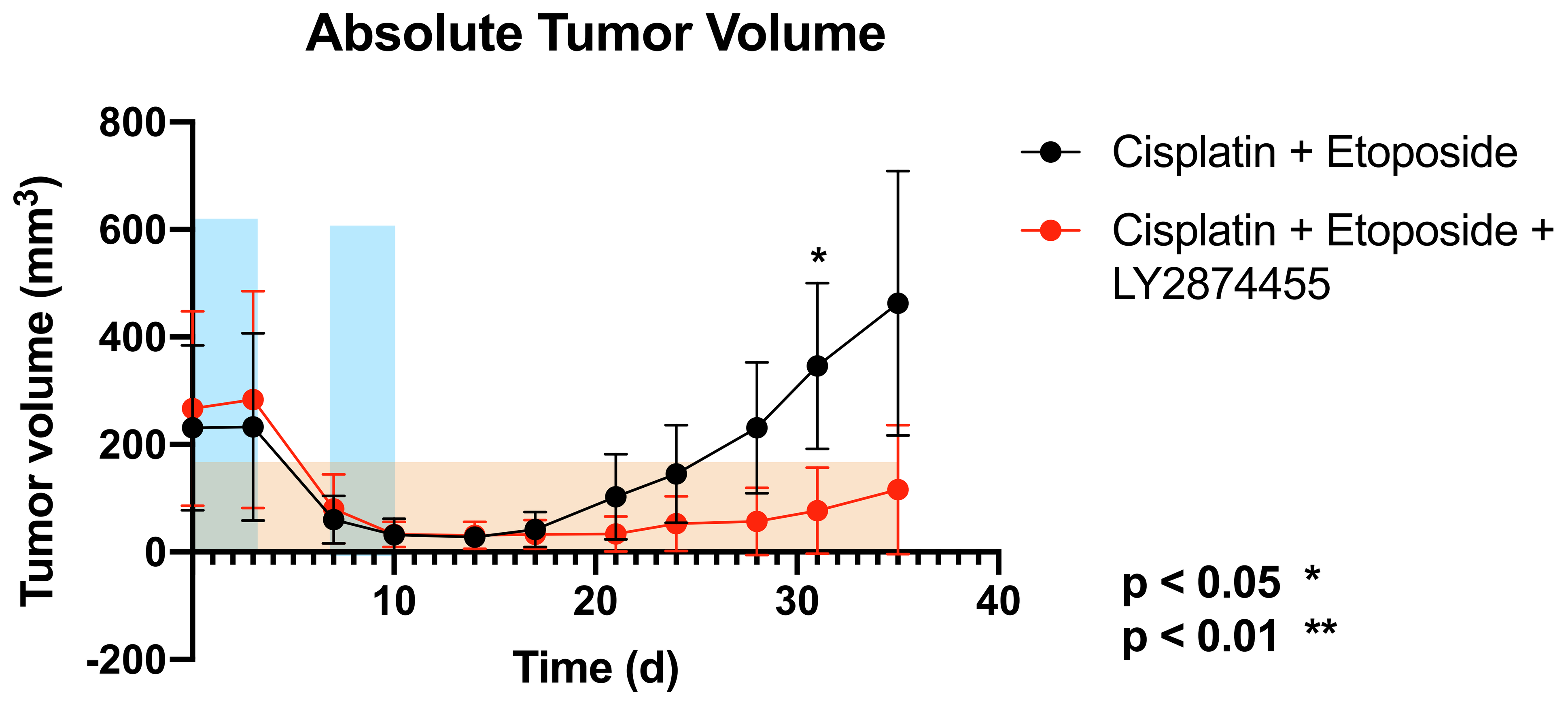

SUPPLEMENTAL INFORMATION

1. Supplemental figure legends
2. Supplemental figures
3. Table S1 – list of diapause DEGs

C1orf210	ENSG00000253313
RPL3	ENSG00000100316
KHDC3L	ENSG00000203908
RAPH1	ENSG00000173166
EMC10	ENSG00000161671
MT1X	ENSG00000187193
ENO1	ENSG00000074800
C19orf25	ENSG00000119559
PSMD8	ENSG00000099341
RINL	ENSG00000187994
TRIM28	ENSG00000130726
WDR92	ENSG00000243667
SNRPD2	ENSG00000125743
RPL18A	ENSG00000105640
PPP2R1A	ENSG00000105568
MYC	ENSG00000136997
PSMD3	ENSG00000108344
RPL36	ENSG00000130255
ARFIP2	ENSG00000132254
ARFIP1	ENSG00000164144
EIF2D	ENSG00000143486
PGLYRP1	ENSG00000008438
ZFPL1	ENSG00000162300
PELO	ENSG00000152684
JMJD4	ENSG00000081692
NSMCE1	ENSG00000169189
ACTN4	ENSG00000130402
MIF	ENSG00000240972
JMJD8	ENSG00000161999
CLDN6	ENSG00000184697
MT1A	ENSG00000205362
CDC34	ENSG00000099804
DDX39B	ENSG00000198563
ARMC5	ENSG00000140691
ARMC6	ENSG00000105676
PPA1	ENSG00000180817
KCTD10	ENSG00000110906
CLPP	ENSG00000125656
GRWD1	ENSG00000105447
TXNIP	ENSG00000265972
MGARP	ENSG00000137463
TELO2	ENSG00000100726
EPHA2	ENSG00000142627
NECAB2	ENSG00000103154
GRB7	ENSG00000141738

CRABP2	ENSG00000143320
AIRE	ENSG00000160224
TWF2	ENSG00000247596
MRPL17	ENSG00000158042
PIK3R2	ENSG00000105647
PIK3R2	ENSG00000268173
ABCB8	ENSG00000197150
MRPL12	ENSG00000262814
PLA2G6	ENSG00000184381
ANAPC10	ENSG00000164162
MRPL20	ENSG00000242485
PRDX2	ENSG00000167815
PRDX5	ENSG00000126432
LDHA	ENSG00000134333
DPH3	ENSG00000154813
PLCG2	ENSG00000197943
NAALAD2	ENSG00000077616
RCE1	ENSG00000173653
TSPAN1	ENSG00000117472
NHEJ1	ENSG00000187736
PRELID1	ENSG00000169230
C19orf53	ENSG00000104979
PMM1	ENSG00000100417
PMM2	ENSG00000140650
MRPL28	ENSG00000086504
PA2G4	ENSG00000170515
PRDX6	ENSG00000117592
DERA	ENSG00000023697
TBL3	ENSG00000183751
SMTN	ENSG00000183963
AKR1B10	ENSG00000198074
EIF6	ENSG00000242372
STAG3	ENSG00000066923
WNK3	ENSG00000196632
OGDH	ENSG00000105953
ID3	ENSG00000117318
REN	ENSG00000143839
DUS1L	ENSG00000169718
ALDH18A1	ENSG00000059573
ANP32B	ENSG00000136938
C12orf75	ENSG00000235162
CD320	ENSG00000167775
ITM2C	ENSG00000135916
PIGO	ENSG00000165282
CITED1	ENSG00000125931

MRPS11	ENSG00000181991
CCDC124	ENSG00000007080
OLA1	ENSG00000138430
MRPL37	ENSG00000116221
ZCCHC17	ENSG00000121766
PANX1	ENSG00000110218
ZCWPW1	ENSG00000078487
TUBA1B	ENSG00000123416
CHAF1A	ENSG00000167670
SMPD1	ENSG00000166311
SDF4	ENSG00000078808
PHGDH	ENSG00000092621
TMED1	ENSG00000099203
SH3BGRL3	ENSG00000142669
EMB	ENSG00000170571
TK1	ENSG00000167900
EIF5AL1	ENSG00000253626
CCT2	ENSG00000166226
SEC13	ENSG00000157020
TPI1	ENSG00000111669
SSR4	ENSG00000180879
GSTO1	ENSG00000148834
MRPS25	ENSG00000131368
TUBB	ENSG00000196230
TFE3	ENSG00000068323
LRRC42	ENSG00000116212
TEX19	ENSG00000182459
MMP12	ENSG00000262406
MRPL52	ENSG00000172590
SLC7A7	ENSG00000155465
PSMA1	ENSG00000129084
RPUSD3	ENSG00000156990
TCP1	ENSG00000120438
ANGPTL4	ENSG00000167772
ALDOA	ENSG00000149925
MRRF	ENSG00000148187
TMEM106A	ENSG00000184988
AHCY	ENSG00000101444
TOMM40	ENSG00000130204
GPS1	ENSG00000169727
SSNA1	ENSG00000176101
HEATR1	ENSG00000119285
CAPG	ENSG00000042493
RBFA	ENSG00000101546
SLC7A3	ENSG00000165349

PSMB6	ENSG00000142507
PSMC3IP	ENSG00000131470
PPP2CA	ENSG00000113575
PSMB4	ENSG00000159377
TUBA3D	ENSG00000075886
ARVCF	ENSG00000099889
PSMB3	ENSG00000277791
STAT4	ENSG00000138378
GPAT2	ENSG00000186281
CCT7	ENSG00000135624
EMC8	ENSG00000131148
EXOSC2	ENSG00000130713
GINS1	ENSG00000101003
MBD3	ENSG00000071655
RANBP1	ENSG00000099901
NPM1	ENSG00000181163
MDH2	ENSG00000146701
WDR18	ENSG00000065268
NR1H2	ENSG00000131408
DOHH	ENSG00000129932
TUBB4B	ENSG00000188229
APTX	ENSG00000137074
BYSL	ENSG00000112578
PPP1CA	ENSG00000172531
PSMC5	ENSG00000087191
PHF5A	ENSG00000100410
TTLL1	ENSG00000100271
ZYX	ENSG00000159840
B9D2	ENSG00000123810
C7orf26	ENSG00000146576
EXOC3L1	ENSG00000179044
ACAA2	ENSG00000167315
GMNN	ENSG00000112312
HDLBP	ENSG00000115677
IPO7	ENSG00000205339
EFTUD2	ENSG00000108883
TATDN3	ENSG00000203705
ABHD12	ENSG00000100997
CCND3	ENSG00000112576
CHCHD3	ENSG00000106554
RUVBL2	ENSG00000183207
ARHGDIA	ENSG00000141522
CFL1	ENSG00000172757
ZNF205	ENSG00000122386
TXNL4A	ENSG00000141759

NUDT14	ENSG00000183828
CA14	ENSG00000118298
TLE4	ENSG00000106829
RPS9	ENSG00000170889
SNRPN	ENSG00000128739
RPSA	ENSG00000168028
THOC3	ENSG00000051596
MAPKAPK3	ENSG00000114738
CCNE1	ENSG00000105173
HGS	ENSG00000185359
PLA2G10	ENSG00000069764
SPNS1	ENSG00000169682
PGP	ENSG00000184207
NHP2	ENSG00000145912
FETUB	ENSG00000090512
KPNB1	ENSG00000108424
SNRPB	ENSG00000125835
PXK	ENSG00000168297
ISYNA1	ENSG00000105655
RNASEH2A	ENSG00000104889
COX16	ENSG00000133983
PSMD14	ENSG00000115233
PSMD13	ENSG00000185627
MRI1	ENSG00000037757
ACACB	ENSG00000076555
PMF1	ENSG00000160783
STIP1	ENSG00000168439
EIF1AD	ENSG00000175376
SYCE1	ENSG00000171772
PODXL	ENSG00000128567
DPP9	ENSG00000142002
NLRP4	ENSG00000160505
ZNF428	ENSG00000131116
EIF4EBP1	ENSG00000187840
CYC1	ENSG00000179091
MAP2K7	ENSG00000076984
TMEM120B	ENSG00000188735
HSPA8	ENSG00000109971
HMGA1	ENSG00000137309
TBCB	ENSG00000105254
UBE2A	ENSG00000077721
NMRAL1	ENSG00000153406
BSPRY	ENSG00000119411
UQCRQ	ENSG00000164405
SYCN	ENSG00000179751

NABP2	ENSG00000139579
PIN1	ENSG00000127445
CCDC130	ENSG00000104957
GPR89A	ENSG00000117262
HSP90AB1	ENSG00000096384
DDX49	ENSG00000105671
GMPPB	ENSG00000173540
MRPL2	ENSG00000112651
SPR	ENSG00000116096
EXO1	ENSG00000174371
TMEM109	ENSG00000110108
SCYL1	ENSG00000142186
BOK	ENSG00000176720
RBM33	ENSG00000184863
GET4	ENSG00000239857
SLC13A2	ENSG00000007216
ITGA3	ENSG00000005884
DNMT3A	ENSG00000119772
CAD	ENSG00000084774
EEF1G	ENSG00000254772
RCN3	ENSG00000142552
PKM	ENSG00000067225
TMEM214	ENSG00000119777
MEP1B	ENSG00000141434
OAS1	ENSG00000089127
DAD1	ENSG00000129562
LCK	ENSG00000182866
DPPA3	ENSG00000187569
ESRP1	ENSG00000104413
DPPA2	ENSG00000163530
AKT1S1	ENSG00000204673
ETF1	ENSG00000120705
MCM5	ENSG00000100297
SLC25A10	ENSG00000183048
EIF4E2	ENSG00000135930
SLC25A5	ENSG00000005022
SLC25A11	ENSG00000108528
SLC25A4	ENSG00000151729
THOP1	ENSG00000172009
FBP2	ENSG00000130957
PLA2G1B	ENSG00000170890
KATNB1	ENSG00000140854
ZBTB45	ENSG00000119574
PSMB10	ENSG00000205220
TBRG1	ENSG00000154144

POLD1	ENSG00000062822
CEACAM21	ENSG00000007129
FSD1	ENSG00000105255
POLR2I	ENSG00000105258
AAMP	ENSG00000127837
CAMK2G	ENSG00000148660
TRIM44	ENSG00000166326
POLR2L	ENSG00000177700
CHGA	ENSG00000100604
GADD45GIP1	ENSG00000179271
CYB5B	ENSG00000103018
RRM2	ENSG00000171848
NAA10	ENSG00000102030
ADRM1	ENSG00000130706
SGTA	ENSG00000104969
FBXL15	ENSG00000107872
GFER	ENSG00000127554
ATOX1	ENSG00000177556
SOD1	ENSG00000142168
BCKDK	ENSG00000103507
EIF3K	ENSG00000178982
PSAT1	ENSG00000135069
ASB6	ENSG00000148331
EIF3G	ENSG00000130811
DRG2	ENSG00000108591
CCM2	ENSG00000136280
GPD1L	ENSG00000152642
P4HB	ENSG00000185624
PFKP	ENSG00000067057
RAN	ENSG00000132341
BCAR1	ENSG00000050820
EIF3B	ENSG00000106263

APP	ENSG00000142192
PIWIL4	ENSG00000134627
GRIK4	ENSG00000149403
AQP3	ENSG00000165272
THG1L	ENSG00000113272
JPH3	ENSG00000154118
PALM	ENSG00000099864
HAVCR1	ENSG00000113249
PSD	ENSG00000059915
CSGALNACT1	ENSG00000147408
STARD4	ENSG00000164211
SLC30A9	ENSG00000014824
ENTPD2	ENSG00000054179
S100A1	ENSG00000160678
SFTPD	ENSG00000133661
EBF1	ENSG00000164330
SHROOM2	ENSG00000146950
FOS	ENSG00000170345
SIRT7	ENSG00000187531
EML1	ENSG00000066629
PATL2	ENSG00000229474
RIPPLY3	ENSG00000183145
ASPG	ENSG00000166183
ACOT1	ENSG00000184227
KDELR3	ENSG00000100196
ANKRA2	ENSG00000164331
CUEDC1	ENSG00000180891
PMEL	ENSG00000185664
SRCAP	ENSG00000282034
SRCAP	ENSG00000080603
KLHL15	ENSG00000174010
ADH1A	ENSG00000187758
FUT11	ENSG00000196968
SDC3	ENSG00000162512
SLC1A1	ENSG00000106688
PRICKLE2	ENSG00000163637
ACCSL	ENSG00000205126
FHIT	ENSG00000189283
TRPM1	ENSG00000134160
SOCS3	ENSG00000184557
PDZD3	ENSG00000172367
GPNMB	ENSG00000136235
HLCS	ENSG00000159267
PLAGL1	ENSG00000118495
LRRC8C	ENSG00000171488

SLC17A5	ENSG00000119899
S100A13	ENSG00000189171
CTNNA3	ENSG00000183230
MSC	ENSG00000178860
ZNF423	ENSG00000102935
BID	ENSG00000015475
PDLIM4	ENSG00000131435
WNT4	ENSG00000162552
GRXCR1	ENSG00000215203
PRRX1	ENSG00000116132
GCH1	ENSG00000131979
AHNAK2	ENSG00000185567
HIP1R	ENSG00000130787
ZBTB10	ENSG00000205189
SOD3	ENSG00000109610
BMP6	ENSG00000153162
MIB1	ENSG00000101752
DAB2	ENSG00000153071
GPR143	ENSG00000101850
FABP3	ENSG00000121769
TMEM98	ENSG00000006042
KBTBD2	ENSG00000170852
TCF4	ENSG00000196628
FXYD6	ENSG00000137726
FGFR2	ENSG00000066468
C1orf116	ENSG00000182795
YPEL2	ENSG00000175155
